# Supplementary figures and images for: A pictural guide to postmortem examination of elephants
Source: PLoS One. 2026 Feb 9;21(2):e0338783. doi: 10.1371/journal.pone.0338783 (PMC12885571; doi:10.1371/journal.pone.0338783)

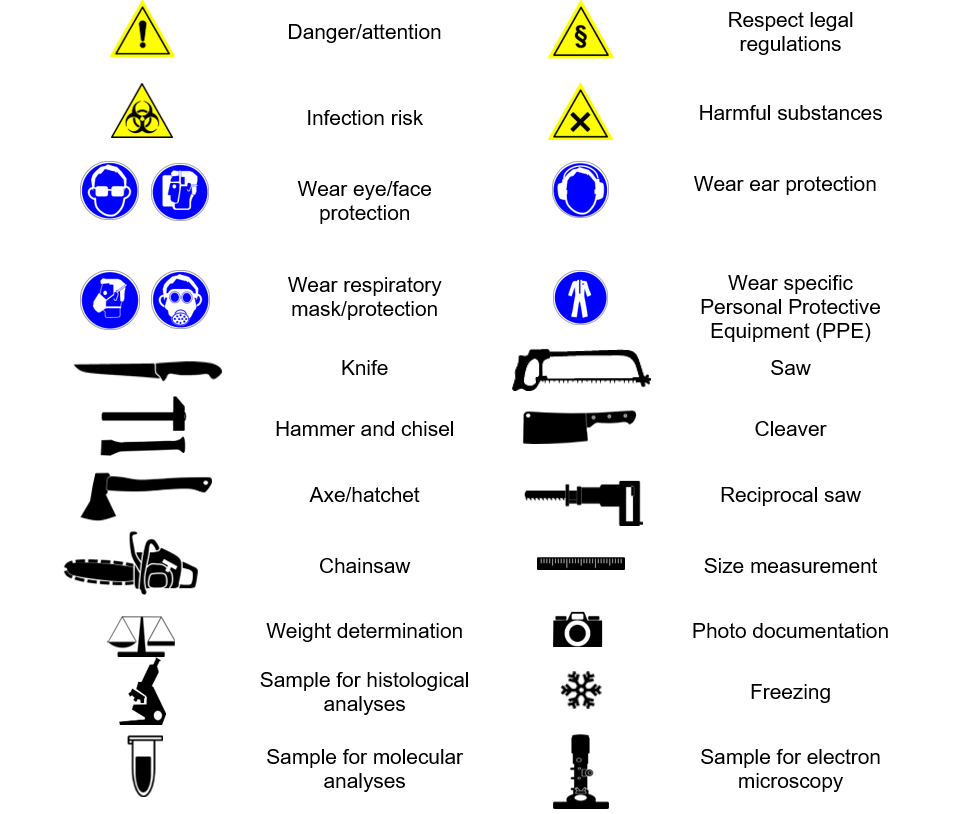

Supplement: S1 Fig — (TIF) [file pone.0338783.s001.tif]
