# Supplementary material for: A pictural guide to postmortem examination of elephants
Source: PLoS One. 2026 Feb 9;21(2):e0338783. doi: 10.1371/journal.pone.0338783 (PMC12885571; doi:10.1371/journal.pone.0338783)
Supplement: S1 File — Standard elephant necropsy form and organ tissue sampling lists. (DOCX) [file pone.0338783.s002.docx]

**S1 Material. Standard elephant necropsy form and organ tissue sampling list.**

**Elephant necropsy - Gross examination**

| **Necropsy date** | |  | | | | **Necropsy number** | | |  |
| --- | --- | --- | --- | --- | --- | --- | --- | --- | --- |
| **Necropsy location** | |  | | | | **Pathologist** | | |  |
| **Contact person/referring veterinarian** | | | | | | **Owner** | | | |
| **Address**  **Phone**  **E-mail** |  | | | | | **Address** | | | |
| **Transferred documents** [original documents/photocopies] | | | | | | | | | |
| **Elephant species**  **□** Asian elephant  □ African elephant | | | **Sex**  □ female □ male | | **Birth date/age** | | | **Elephant name** | |
| **Transponder number** | | | | **ISIS-number^1^** | | | **Studbook-number** | | |
| **Body weight (kg)**  [□ reported □ actual □ estimated] | | | | | | | | | |
| **Death date/Location/PMI** | | | | | | | | | |
| **TB-status □ positive □ suspected □ unknown/not tested □ negative** | | | | | | | | | |
| **Form of death □ naturally/found dead □ other**:  **□ euthanized** [drug, application, dose]: | | | | | | | | | |
| **Number of elephants in herd** | | | | | | **Other diseased elephants** | | | |
| **Collection of *intra vitam* blood/serum samples**  **□ no □ yes** [type, volume, storing location]**:** | | | | | | | | | |

^1^International Species Inventory System

| **Requested examinations/questions** | **□ Routine gross- and histo-pathology**  **□ Suspected intoxication**  **□ Forensic case** |
| --- | --- |
| **Requested sample collection for third parties** [please, list here and attach respective documents] | |
| **Clinical/Case history** [TB-status in herd, housing, diet, social status in herd, number of past births, last delivery, patient history, clinical and lab-findings (list and attach documents), circumstances of death] | |

| **External examination** | | | | |
| --- | --- | --- | --- | --- |
| **Dismemberment & “Locomotion “Team** | **Signs of death: Degree of autolysis:** | | | |
|  |  | | | **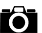** |
|  | **Identification** [transponder, tattoos, distinguishing features]**:** | | |  |
|  | **Physical condition:**  **Nutritional status:**  **Age estimation:** | | |  |
|  |  | | **Sampling^2^** | **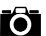** |
|  | **Skin** | |  |  |
|  | **Mammary gland** | |  |  |
|  | **Feet** [toenails, sole]**:** | |  |  |
|  | **Body orifices** | **Eyes** |  |  |
|  |  | **Ears** |  |  |
|  |  | **Mouth** |  |  |
|  |  | **Trunk-nostrils** |  |  |
|  |  | **Anus** |  |  |
|  |  | **Vulva** |  |  |
|  |  | **Prepuce, Penis** |  |  |
|  | **Superficial lymph nodes**  (mandibular, superficial cervical, popliteal, superficial inguinal) | |  |  |

^2^Indicate sample type: Microbiology (bacteriology, virology, mycology), parasitology, toxicology, histology, electron-microscopy, cryohistology, molecular analyses.

| **Musculoskeletal system** | | **Sampling** | **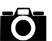** |
| --- | --- | --- | --- |
| **Dismemberment & ”Locomotion”Team** | **Joints** [hip, knee, shoulder, elbow, feet] |  |  |
|  | **Musculature** |  |  |
|  | **Bones** |  |  |
|  | **Bone marrow** |  |  |
|  | **Feet** [after X-ray/CT/MRI-imaging, if appropriate] |  |  |

| **Body cavities and thorax**  [effusions, situs, serosae, adipose tissue stores, lymph nodes] | | **Sampling** | **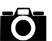** |
| --- | --- | --- | --- |
| **GIT Team** | **Abdominal cavity** |  |  |
|  | **Pelvic cavity** |  |  |
| **Thorax Team** | **Thorax** |  |  |

| **Urogenital system** | | **Sampling** | **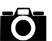** |
| --- | --- | --- | --- |
| **UGT Team** | **Kidneys** |  |  |
|  | **Ureter, bladder, urethra** |  |  |
|  | **Testis (2), epididymis (2)**  **Prostate, seminal vesicles, bulbourethral gland** |  |  |
|  | **Penis/male urethra** |  |  |
|  | **Ovary (2), salpinx (2)** |  |  |
|  | **Uterus** |  |  |
|  | **Gestation**  **□ no**  **□ yes**  [Fetus, size, weight, position, placenta/embryonic membranes, umbilical cord]**:** |  |  |
|  | **Cervix, vulva, clitoris** |  |  |
| **“Loco-motion”Team** | **Mammary gland** |  |  |

| **Digestive system and abdominal lymphatic organs** | | **Sampling** | **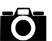** |
| --- | --- | --- | --- |
| **Head Team** | **Mouth, teeth** |  |  |
| **GIT Team** | **Tongue** [handed over from Thorax-Team] |  |  |
|  | **Esophagus** [handed over from Thorax-Team] |  |  |
|  | **Spleen** |  |  |
|  | **Stomach** |  |  |
|  | **Small intestine** |  |  |
|  | **Caecum** |  |  |
|  | **Large intestine** |  |  |
|  | **Rectum** |  |  |
|  | **Liver** |  |  |
|  | **Pancreas** |  |  |
|  | **Mesentery/lymph nodes** |  |  |

| **Respiratory system and thoracic lymphatic organs** | | **Sampling** | **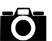** |
| --- | --- | --- | --- |
| **Thorax Team** | **Trunk-passages** [trunk handed over from Head-team] |  |  |
|  | **Pharynx, larynx** |  |  |
|  | **Retropharyngeal lymph nodes** |  |  |
|  | **Trachea** |  |  |
|  | **Bronchi** |  |  |
|  | **Lungs** |  |  |
|  | **Lymph nodes** (tracheobronchial, pulmonary) |  |  |
|  | **Thymus** |  |  |
|  | **Mediastinum** |  |  |

| **Cardiovascular system** | | **Sampling** | **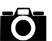** |
| --- | --- | --- | --- |
| **Thorax Team** | **Pericardium** |  |  |
|  | **Heart** [epicardium, myocardium, endocardium, valves, great vessel-trunks] |  |  |
|  | **Collection of *post mortem* blood** |  |  |
|  | **Great vessels** [thoracic and abdominal aorta, great veins] |  |  |

| **Nervous system** | | **Sampling** | **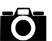** |
| --- | --- | --- | --- |
| **Head & Dismemberment Team** | **Brain** |  |  |
|  | **Meninges** |  |  |
|  | **Spinal cord** [indicate segments] |  |  |
|  | **Peripheral nerves** |  |  |

| **Endocrine system** | | **Sampling** | **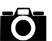** |
| --- | --- | --- | --- |
| **Head Team** | **Pituitary** |  |  |
|  | **Thyroid, parathyroid** |  |  |
| **UGT** | **Adrenal glands** |  |  |

| **Sensory organs** | | **Sampling** | **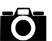** |
| --- | --- | --- | --- |
| **Head Team** | **Eyes** |  |  |
|  | **Ears** [pinnae, external ear canal, middle ear, inner ear (optionally)] |  |  |
|  | **Vomeronasal (Jacobson's) organ** |  |  |

**Additional comments/observations**

**Summary of preliminary findings/ morphologic diagnoses**

**Elephant necropsy - Organ/tissue sample list**

| - *Prepare/process samples according to the instructions of the other necropsy-teams.* - *Label samples appropriately with ID, organ/tissue, sample-type, fixative, and storage.* - *Document generated samples in list (indicate sample type and sample numbers).* - *Process samples for molecular analyses first (prevent sample degradation).* - *Communicate any missing samples with the responsible pathologist before the necropsy ends.* |
| --- |

| **Organ/tissue sample list**  [specify organ/tissue, sample type, sample number/volumes and recipient] | | | | | | | |
| --- | --- | --- | --- | --- | --- | --- | --- |
| **Organ/tissue** | **MB/P** | **TOX** | **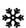** | **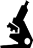** | **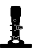** | **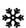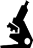** | **Designation** |
| **Adrenal** |  |  |  |  |  |  |  |
| **Aorta** |  |  |  |  |  |  |  |
| **Blood/Serum** |  |  |  |  |  |  |  |
| **Bone/Bone marrow** |  |  |  |  |  |  |  |
| **Brain** |  |  |  |  |  |  |  |
| **Bronchus** |  |  |  |  |  |  |  |
| **Bulbourethral gland** |  |  |  |  |  |  |  |
| **Caecum** |  |  |  |  |  |  |  |
| **Diaphragm** |  |  |  |  |  |  |  |
| **Epididymis** |  |  |  |  |  |  |  |
| **Esophagus** |  |  |  |  |  |  |  |
| **Eye** |  |  |  |  |  |  |  |
| **Heart** |  |  |  |  |  |  |  |
| **Hemal node** |  |  |  |  |  |  |  |
| **Kidney** |  |  |  |  |  |  |  |
| **Large intestine** |  |  |  |  |  |  |  |
| **Liver** |  |  |  |  |  |  |  |
| **Lung** |  |  |  |  |  |  |  |
| **Lymph nodes** |  |  |  |  |  |  |  |
| **Mammary gland** |  |  |  |  |  |  |  |
| **(Skeletal) Muscle** |  |  |  |  |  |  |  |
| **Nerve** |  |  |  |  |  |  |  |
| **Organ/tissue** | **MB/P** | **TOX** | **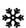** | **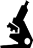** | **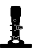** | **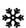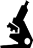** | **Designation** |
| **Ovary** |  |  |  |  |  |  |  |
| **Pancreas** |  |  |  |  |  |  |  |
| **Parathyroid** |  |  |  |  |  |  |  |
| **Penis** |  |  |  |  |  |  |  |
| **Prepuce** |  |  |  |  |  |  |  |
| **Pituitary** |  |  |  |  |  |  |  |
| **Prostate** |  |  |  |  |  |  |  |
| **Salivary gland** |  |  |  |  |  |  |  |
| **Seminal vesicle** |  |  |  |  |  |  |  |
| **Skin** |  |  |  |  |  |  |  |
| **Small intestine** |  |  |  |  |  |  |  |
| **Spinal cord** |  |  |  |  |  |  |  |
| **Spleen** |  |  |  |  |  |  |  |
| **Stomach** |  |  |  |  |  |  |  |
| **Temporal gland** |  |  |  |  |  |  |  |
| **Testis** |  |  |  |  |  |  |  |
| **Thymus** |  |  |  |  |  |  |  |
| **Thyroid** |  |  |  |  |  |  |  |
| **Tongue** |  |  |  |  |  |  |  |
| **Tonsil** |  |  |  |  |  |  |  |
| **Trachea** |  |  |  |  |  |  |  |
| **Trunk** |  |  |  |  |  |  |  |
| **Ureter** |  |  |  |  |  |  |  |
| **Urinary bladder** |  |  |  |  |  |  |  |
| **Uterus** |  |  |  |  |  |  |  |
| **Vagina/Cervix** |  |  |  |  |  |  |  |
|  |  |  |  |  |  |  |  |
|  |  |  |  |  |  |  |  |
|  |  |  |  |  |  |  |  |
|  |  |  |  |  |  |  |  |
|  |  |  |  |  |  |  |  |
|  |  |  |  |  |  |  |  |
|  |  |  |  |  |  |  |  |
|  |  |  |  |  |  |  |  |
|  |  |  |  |  |  |  |  |

| **Sample types** | | **Symbol** | **Sampling advice** |
| --- | --- | --- | --- |
| **Micro-biology** | **Bacteriology, Virology, Mycology** | **MB-B,**  **MB-V,**  **MB-M** | Take sterile culture swabs (s) or fresh tissue (t) samples in culture dish. |
| **Parasitology** | | **P** | Take appropriate volume of fresh tissue/ingesta (200g) and fill in sterile sample container. |
| **Toxicology** | | **TOX** | Take appropriate volume of fresh tissue/ingesta (500 ml/500 g) or vitreous (10 ml) and fill in sterile sample container. Take duplicate samples. Store at RT or freeze according to the scheduled downstream toxicology-analyses |
| **Histology** | | **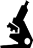** | Fix tissue samples (max. 2 cm thick) in neutrally-buffered 4% formaldehyde solution (tissue:fixative = 1:10). If necessary, lamellate thick tissue/organ samples prior to fixation and change fixation solution after few hours. |
| **Electron microscopy** | | **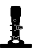** | Fix tissue pieces (max. 2 x 2 x 2 mm³) in 3% buffered glutaraldehyde solution |
| **Cryo-histology** | | **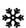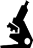** | Block tissue samples (10 x 5 x 5 mm³) in mounting medium in freezing bowls and freeze in liquid-nitrogen-cooled isopentane (-150°C), store at -80°C. |
| **Frozen** | | **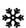** | Indicate freezing/storage-Temperature (-20°C, -80°C, -196°C). |
| **Molecular/biochemical analysis** | | **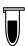** | Freeze on dry-ice, place in cryo-cup and store at -80°C. For long-term storage of samples, store at -150°C. |
