# Supplementary material for: A pictural guide to postmortem examination of elephants
Source: PLoS One. 2026 Feb 9;21(2):e0338783. doi: 10.1371/journal.pone.0338783 (PMC12885571; doi:10.1371/journal.pone.0338783)
Supplement: S2 File — Checklist for the preparation and organization of an elephant necropsy with work instructions (handouts) for elephant necropsy teams/personnel. (DOCX) [file pone.0338783.s003.docx]

**S2 Material. Checklist and work-instructions for the organization and performance of an elephant necropsy.**

**Checklist for the pathologist in charge**

**for the planning/organization of an**

**elephant necropsy**

**1. Upon announcement of the imminent necropsy of an elephant clarify the following points with the referring veterinarian/zoo/circus:**

- Note **contact data/phone numbers.**
- Obtain **clinical/case history.**
- Inquire **TB-status** of elephant / herd.
- Ask referring veterinarian/owner to provide all necessary **documents (ISIS-N°, studbook-N°)**
- Ask referring veterinarian to **arrange the transport of the elephant to the necropsy site.**
- Provide detailed **directions** to the necropsy site and relevant details on the **accessibility** (one-way roads, **vehicle size/weight restrictions**)
- Exchange contact data/mobile phone numbers with the transport personnel
- Expected **delivery date/time**? Try to **keep PMI short**, if possible, euthanize shortly before transport to necropsy site. If necessary (warm weather, long haul), **refrigerate the dead elephant with icebags** (1000-2000 kg), placed around the abdomen.
- Ask referring veterinarian to **collect *intra vitam* blood/serum samples** (10 x 10 ml), if possible.
- Drug/dose applied for euthanasia?
- Ask referring veterinarian to provide a **complete list of tissue samples to be collected for third parties** in advance of the necropsy (detailed list with organ/tissue, sample location, sample numbers, fixation, storage temperature) including the complete contact data of the sample recipients.
- If the referring veterinarian(s) want(s) to join the necropsy, ask for their clothing sizes to provide them with **appropriate PPE** and inform them about **applicable workplace safety and infection protection regulations**.

**2. Organization of prearrangements of the necropsy**

- **Reserve the necropsy room**/premises for the elephant necropsy on the scheduled date/time. Estimate a time requirement of a complete day (>8 hours).
- **Inform available personnel/helpers on the upcoming elephant necropsy** and collect their contact data/phone numbers and clothing sizes to provide them with appropriate PPE.
- Organize the **assignment of the personnel to the different necropsy teams/tasks** (**S2 Material Table**, below), considering the large animal necropsy experiences of the single persons. Inform staff about their team affiliation, team members, and tasks in advance. **Nominate qualified first aiders** (**S2 Material Table**, below).
- Organize the availability of the required **PPEs** (protective clothing, disposable, water-repellent coveralls with hoods, gloves, respiratory masks, PAPRs (Powered Air Purifying Respirators), eye protective glasses/face shields, etc., according to **Section 4**), **with sufficient replacement capacities**.
- Organize the availability of all **required necropsy instruments**, including “special” tools, such as chain saw, axes, metal detectors (if appropriate), *etc.*, and **equipment used for the transportation of elephant body parts**, according to (**S1 Table**).
- Organize the availability of all required equipment for **photo documentation** (**S1 Table**).
- If appropriate, **contact potential third-party recipients of elephant organ/tissue samples and ask them to provide detailed lists of tissue samples to be collected**, as soon as possible (determine a deadline).

Use the sample lists for third-party sample recipients and the “own” sample list to **determine a preliminary list of organ/tissue samples** to be collected (**S1 Material**).

- Organize the availability of the accordingly required amount of **sampling supplies**, such as containers, sterile instruments, culture swabs, fixatives, ice, dry ice, liquid nitrogen, *etc.*, according to (**S1 Table**).
- Correspondingly organize the correct **labeling** **of sample containers**, boxes, etc. prior to the necropsy, if possible.
- If performance of **imaging techniques such as X-ray, CT, or MRI** is scheduled on (separated) elephant body parts, **contact the respective local institutions/clinics and arrange appointments** correspondingly. Inform on possible TB-risk and discuss the package/ wrapping of elephant body parts during the transport and the acquisition of X-ray, CT, or MRI images. Take down the contact data/phone number of the corresponding contact person(s) and designate a person in charge for the transport of elephant body parts to the clinic, *e.g.,* from the “Locomotion” necropsy team.
- Organize the **collection of the carcass/animal waste** (*est.* 4-8 m³) on the subsequent day by a certified rendering plant.
- Plan the **allocation of workspaces for the different necropsy teams** within the available necropsy premises (provide a sketch, as exemplified in **S2 Material Figure**, below) and provide this to the staff preparing the necropsy room. **Prepare adapted work instructions for the separate necropsy teams** (included in **S2 Material**, below). **Prepare the necropsy room accordingly and provide each workspace with the appropriate necropsy instruments, equipment, and a printout of work instructions.**
- **Formulate and list applicable specific safety and health regulations** for the elephant necropsy adapted to the present circumstances (**Section 4**).
- Adapt the necropsy form to the given circumstances/sampling lists together with the person designated as “Secretary”.
- **Print out the reviewed/adapted necropsy forms, sample lists, work space allocation sketches, contact details, and applicable technical work instructions. Display one set of printouts on a notice board inside the necropsy room visible for all personnel.**
- Organize the appropriate **final cleaning and disinfection** of the necropsy instruments, equipment, and premises (provide sufficient volumes of disinfectant solution), and the correct disposal of any additional necropsy waste (used disposable PPEs and sample plates etc.).

**3. Management of the necropsy**

- **Check the appropriate preparation of the necropsy room, the completeness of the required instruments, equipment, sampling-materials, supplies, and PPE, and the functionality of eye showers.**
- **Assemble all staff and explain the scheduled sequence of the necropsy** from beginning to end, and the **tasks**, and **assigned workspaces of the different necropsy teams** and their members, and **contact-persons** (head, secretary, photographer, stand-in men).
- **Explain/repeat the sampling process**: Lesions are identified by the necropsy teams who also arrange the photo documentation (*i.e.,* call the “photographer”). Lesions, as well as the scheduled organ/tissue samples are then sampled (as indicated in the provided work instructions for the separate necropsy teams), appropriately labeled and handed to the “Sampling”-team: The tissue sample(s) is/are placed on disposable paper bowls/plates and labeled with a waterproof pen, the “Sampling”-team processes the samples further.
- **Remind staff not to dispose any organs/tissues until prompted to** (ensure the completion of the sampling process).
- **Repeat and explain the defined workplace safety and health protection rules** (PPE, zoonotic potential, keeping proper distance from persons operating axes/chainsaws, *etc.*), show the location of the first aid cabinets, eye showers, and the telephone, and get confirmation of the instruction by signatures (especially non-resident helpers/ “necropsy-guests”).
- **Clarify all remaining open questions** of staff members before starting with the necropsy.
- **During the necropsy: Pay attention to compliance with the set workplace safety and health protection rules**. Be available for questions of staff members. Keep an overview on the sampling procedures/special examinations (X-ray/CT/MRI).
- **Control the complete and correct extraction, processing, and documentation of all samples** (check against the sampling lists) and the proper acquisition of **photo images**, and the complete **documentation of all gross pathological findings**, before releasing the carcass/organ waste for disposal.
- **Document any transfer of legally protected elephant body parts** (e.g., tusks) appropriately (signed attestation with date).
- Attach all necessary **legal documents** to the histology report.
- Remind the **personnel involved in the further processing of the samples** (*e.g.,* next day-sectioning of fixed tissue samples) to **wear appropriate eye and respiratory protection** (TB-risk).
- Ensure the correct **labeling and shipment of organ/tissue samples for third-party sample recipients**.
- [Thank all helpers].

| **S2 Material Table: Contact data and team affiliations of elephant necropsy staff** | | | |
| --- | --- | --- | --- |
| **Necropsy date:** | | ***direct questions to*:**    **E-mail:**  **Phone:** | |
| **Location/meeting point:** | |  |  |
| **Time:** | |  |  |
| **Team** | **Name** | **Phone** | ***Confirm briefing on safety and health instructions (signature)*** |
| **Superintendent** |  |  |  |
| **Secretary** |  |  |  |
| **Photographer** |  |  |  |
| **First aiders** |  |  |  |
|  |  |  |  |
| **Knife sharpener** |  |  |  |
| **Stand-in/spare man** |  |  |  |
| **-Dismemberment** |  |  |  |
|  |  |  |  |
|  |  |  |  |
|  |  |  |  |
| **-Gastrointestinal** |  |  |  |
|  |  |  |  |
|  |  |  |  |
|  |  |  |  |
| **-Locomotion** |  |  |  |
|  |  |  |  |
|  |  |  |  |
| **-Thorax** |  |  |  |
|  |  |  |  |
| **-Head** |  |  |  |
|  |  |  |  |
| **-Urogenital** |  |  |  |
|  |  |  |  |
| **-Sampling** |  |  |  |
|  |  |  |  |
|  |  |  |  |
| **-Waste disposal**  **& disinfection** |  |  |  |
|  |  |  |  |

| **Contact person(s) for X-ray, CT, or MRI imaging of elephant body parts/samples** | | | | |
| --- | --- | --- | --- | --- |
| **Imaging/sample** | **Location** | **Appointed time** | **Contact person** | **Phone** |
|  |  |  |  |  |
|  |  |  |  |  |
|  |  |  |  |  |
|  |  |  |  |  |

**S2 Material Figure: Example for a sketch displaying the allocation of workspaces for the different necropsy teams, adapted to the available premises.**


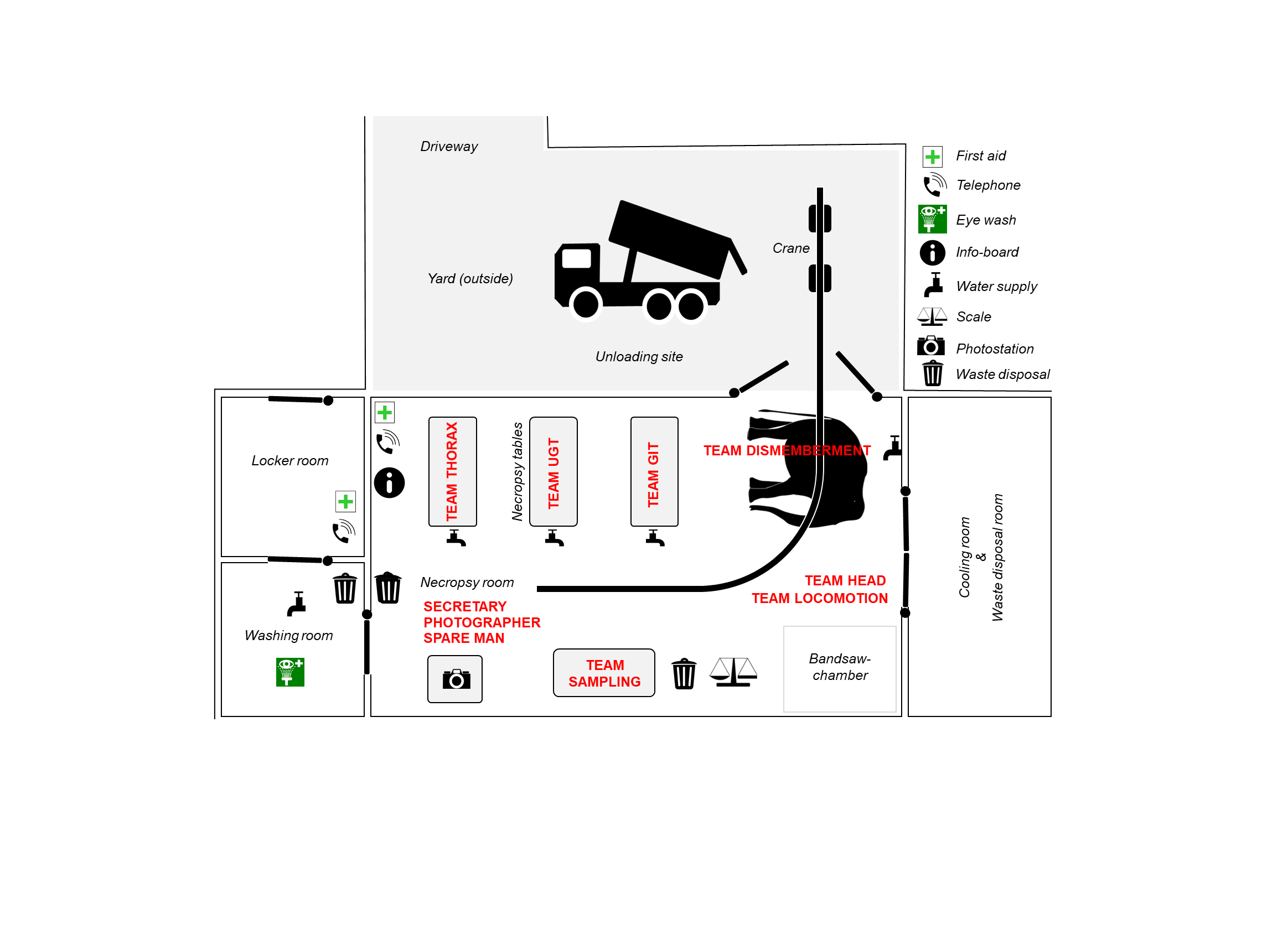


*The sketch is provided to the personnel preparing the necropsy room in advance of the necropsy to arrange the necropsy “furniture” (desks, trash bins, photo station, scale, etc.) and distribute the appropriate necropsy instruments and sampling supplies to the different necropsy team stations. The sketch should be made available to all participants of the necropsy in advance and also be displayed inside the necropsy room, e.g., on the info board. It should also indicate the positions of first aid equipment, telephone(s) and eye wash stations.*

**Work instructions (handouts) for elephant necropsy teams/personnel**

*Copy the handout work instructions and provide separate necropsy teams to be at hand in the necropsy room. Fill in specific instructions for sampling/collection of organ/tissue samples. The work-instruction for the “Sampling” team is included in the Elephant necropsy - Organ/tissue sample lists (***S1 Material***).*

**Work instructions for the “Secretary”**


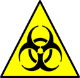

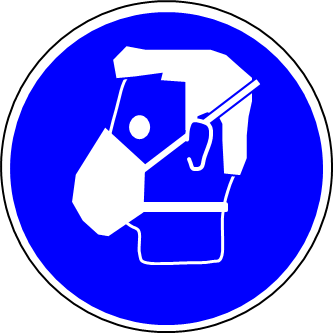


*The secretary is responsible for the appropriate documentation of all relevant paperwork (documents, forms, protocols), directs the necropsy procedure and ensures completeness of all collected samples.*

**Before the necropsy:**

- Support the pathologist in charge with the **organization and planning of the necropsy**. **Prepare, fill in, copy, and distribute all documents, forms, lists, contact information relevant for the necropsy**. Help to prepare the necropsy room and provide the “handout”-work instructions to the different necropsy teams/staff.

**During the necropsy:**

- **Maintain the overview on the current state of the necropsy process** and the progress of the collection of organ/tissue samples.
- Follow the ongoing steps in the sequence of the necropsy procedure and **move from team to team to take notes, record gross findings, and document what organs/tissues are sampled**.
- **Be in steady contact** with the pathologist in charge, the photographer, the sampling and necropsy teams to communicate any upcoming problems/delays.
- **Direct/assign the occupation of the spare man.**

**At the end of the necropsy**

- **Get feedback from all necropsy teams/staff** to control the completion of all necropsy steps and imaging techniques (X-ray, CT, MRI), the recording of all findings, the completeness and correct processing of all samples and photo-images. Report to the pathologist in charge **before authorizing the disposal of the carcass/remains** and dismissal of the necropsy staff.
- **Collect all sample lists/notes from the necropsy teams.**

**After the necropsy**

- Support the pathologist in charge with the **writing of the report**, the **animal waste disposal paperwork**, the **distribution/shipment of organ/tissue samples** to third-party sample recipients, the **documentation of results of initiated analyses** (microbiology, toxicology, etc.), and the correct **further processing of organ/tissue samples** (sectioning, embedding, etc.).

| **Tick box** | **Document/Form/List** | **Comments** |
| --- | --- | --- |
| 🞎 |  |  |
| 🞎 |  |  |
| 🞎 |  |  |
| 🞎 |  |  |
| 🞎 |  |  |
| 🞎 |  |  |
| 🞎 |  |  |
| 🞎 |  |  |
| 🞎 |  |  |

**Work instructions for the “Spare man”**


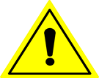

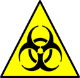

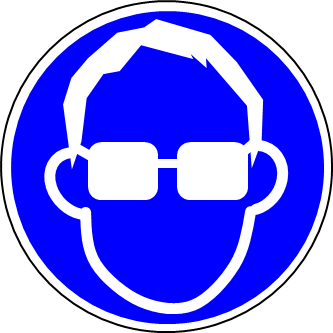

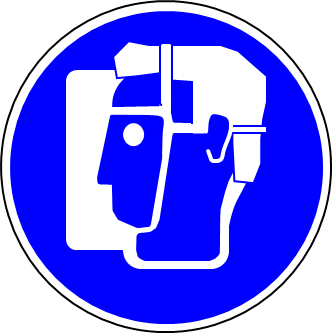

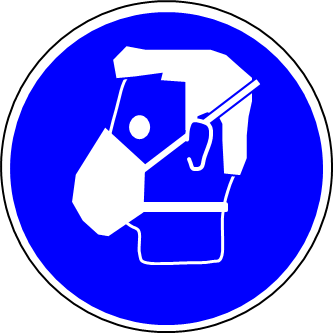

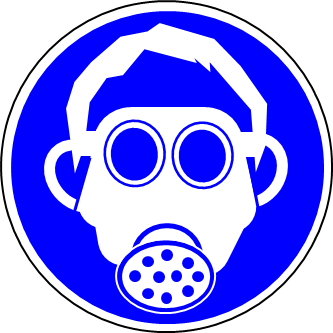

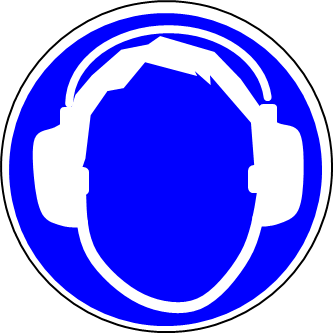

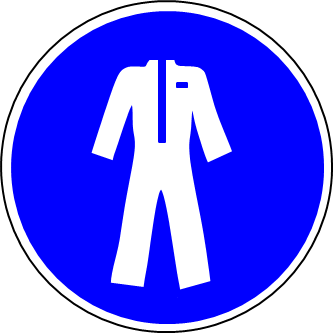

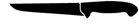


- The occupation of the spare man is assigned by the secretary.
- Be available for support of other personnel with short errands and assistance (e.g., restock consumables, duct-tape gloves of other members, etc.).
- Be available for “in-house” or “on-campus” transports of samples to other institutes, if applicable.
- Mind the workplace/health-safety regulations of the different necropsy teams/activities.

**__________________________________________________________**

**Work instructions for the “Knife sharpener”**


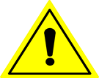

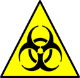

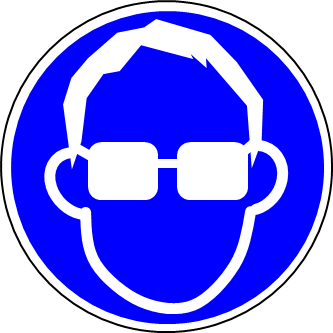

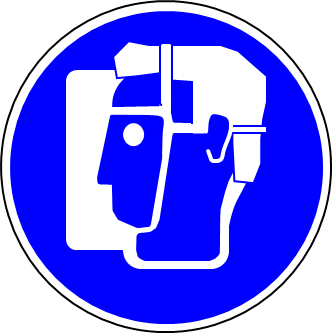

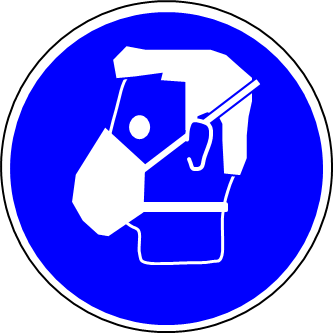

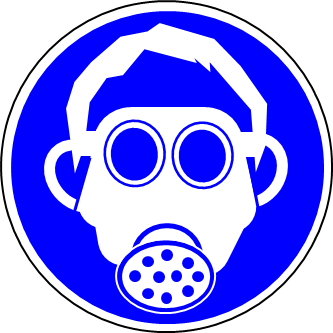

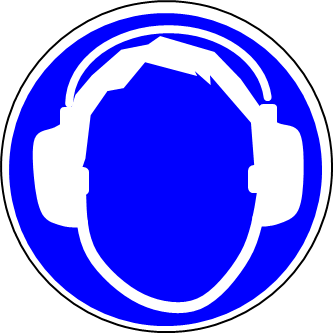

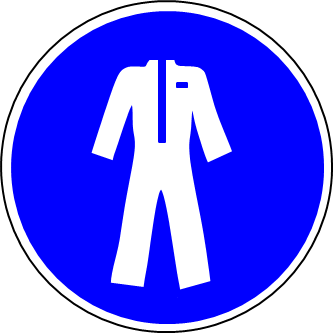

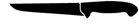


- Constantly collect, clean, sharpen, and return used, blunted knives from the necropsy teams.
- Mind the workplace/health-safety regulations of the different necropsy teams/activities.

**__________________________________________________________**

**Work instructions for team “Waste and disinfection”**


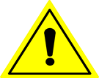

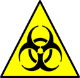

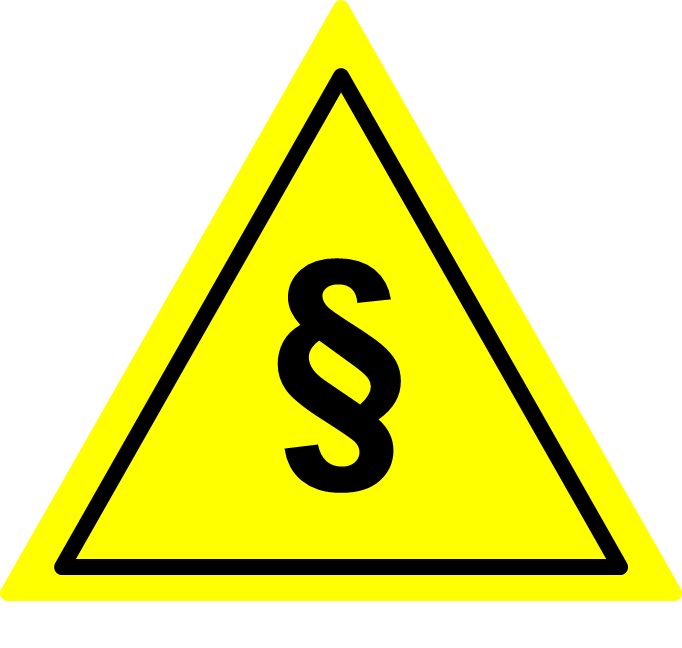

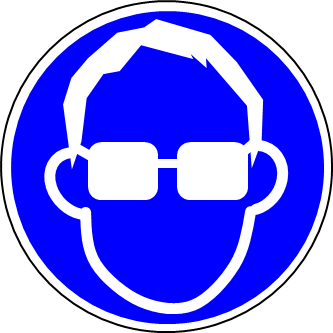

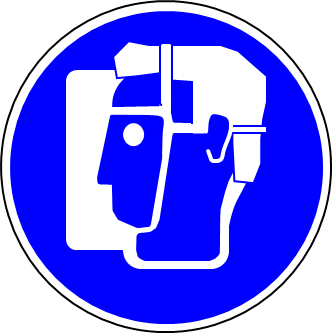

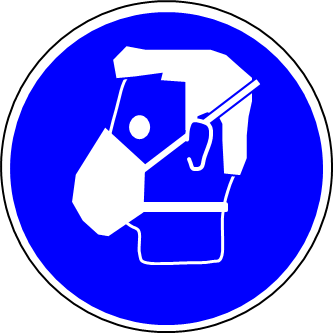

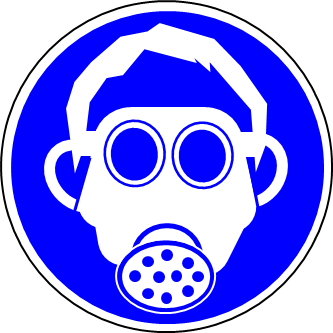

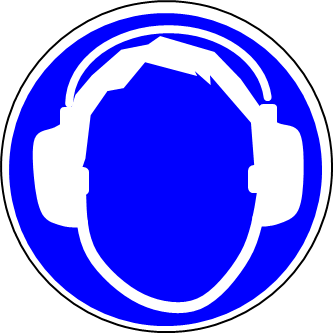

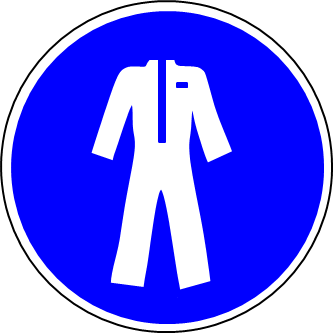

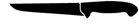

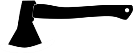

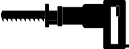

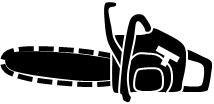


- **Always wear adequate PPE** (respiratory masks, eye protection, *etc*.).
- **Do not dispose of any elephant body parts before approval by the pathologist in charge**.
- **Dismantle the carcass** to allow the transport of body parts (should fit into the waste container).
- **Inform the pathologist in charge of additional (yet undiscovered) pathological findings.**
- Dispose animal waste appropriately, **store safely and refrigerated** (if possible) until collection by a certified rendering plant.
- **Do not hand over any elephant body parts to unauthorized persons**.
- **Transfer of any elephant body parts** (*e.g.,* rendering plant) must be **documented and receipted**.
- Appropriately dispose used disposable PPE, necropsy and sampling supplies.
- **Use officially approved tuberculocidal disinfection solutions** for the disinfection of instruments, equipment and rooms, according to the recommendations provided by the manufacturer.

**__________________________________________________________**

**Work instructions for the “Photographer”**


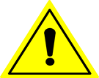

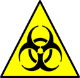

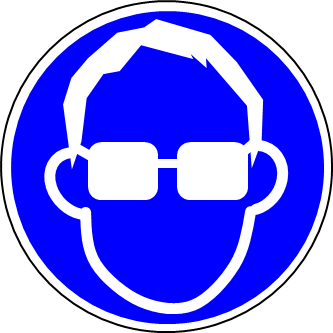

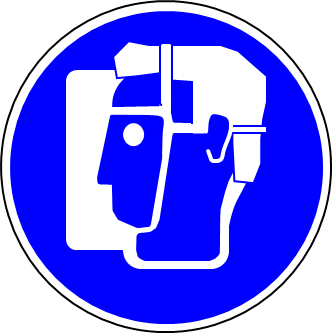

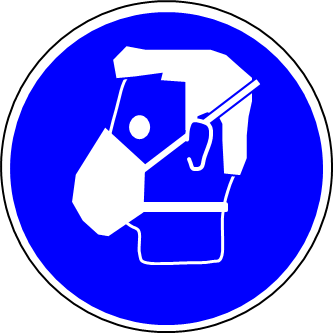

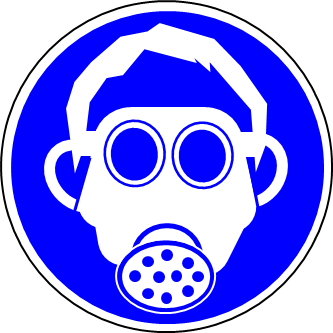

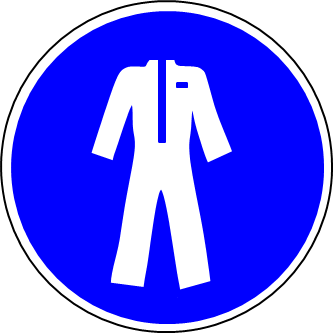

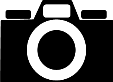

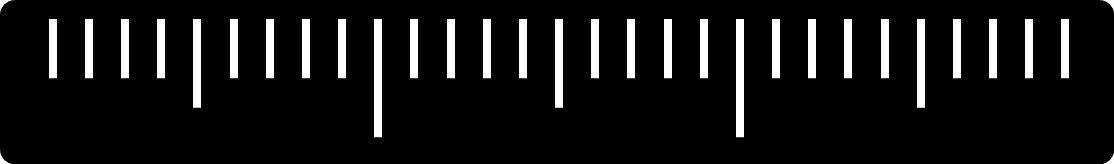


The photographer is responsible for the appropriate photo documentation of animal identity (forensic cases) and necropsy gross findings.

**In advance of the necropsy**:

- **Prepare and control the necessary photo-equipment** [camera (digital) with normal and close-up lenses, replacement memory cards and charged replacement batteries (!), repro-stand, tripod, photo-pads (waterproof, washable, smooth, non-reflective, in white, grey and black), reference scales (writable) for different sample sizes, waterproof pen, paper towels, replacement gloves]
- **Study the** scheduled sequence of the necropsy-process and compare this to the **list of distinct image contents to be photographed in any case** (*e.g.,* to document the identity of the elephant or externally visible injuries), which is provided by the superintendent/pathologist in charge, to be ready to take the photos at the right moment(s).

**During the necropsy**

- **Stand ready to photograph gross lesions** identified by the different necropsy teams at their respective working stations/places (often the body parts are too heavy to move to the photo-station).
- **Take multiple overview- and detail images from each lesion/object**.
- **Always use an appropriately sized ruler**.
- **Each image must contain a** **label with information on the animal/necropsy-ID and the depicted organ/tissue**.


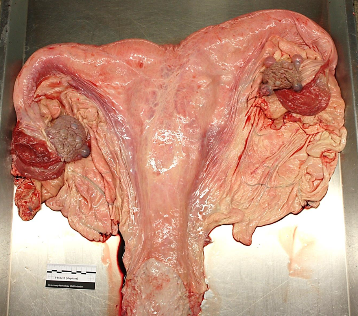


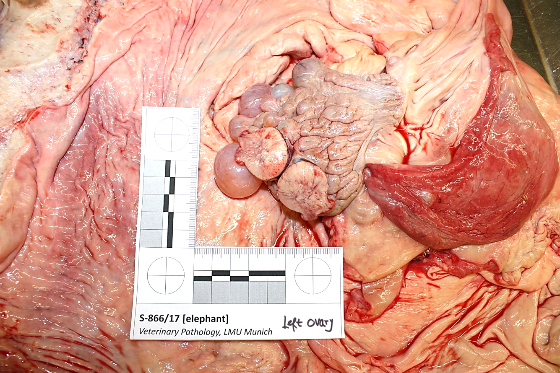


*Example for adequate taking of pictures: Acquire overview (left) and detail (right) -images with appropriately sized rulers and labels. [Here: Uterus and ovary from an aged female Asian elephant.]*

- To increase the depth of field in close-up images, use high apertures (f-numbers), appropriate illumination and adapted (long) exposure times (implying the **use of tripods or repro-stands** to avoid blurred images).
- Try to avoid (unnecessary) “bloody” backgrounds, **remove saw-chips, splinters, hairs and dirt (etc.) from the surface of the imaged sample**.
- If imaging cut surfaces of organs/tissues, avoid irregular or jagged sections/incisions (get clean, straight-cut edges/planes).
- After imaging of a sample, ensure that it is passed to the sampling team.
- **Protocol all taken images in the image list.**

| **List of gross images** | | | | | |
| --- | --- | --- | --- | --- | --- |
| **Necropsy number:** | | | **Date:** | | |
| **Organ/lesion** | ***overview*** | ***detail*** | **Organ/lesion** | ***overview*** | ***detail*** |
|  | 🞎 | 🞎 |  | 🞎 | 🞎 |
|  | 🞎 | 🞎 |  | 🞎 | 🞎 |
|  | 🞎 | 🞎 |  | 🞎 | 🞎 |
|  | 🞎 | 🞎 |  | 🞎 | 🞎 |
|  | 🞎 | 🞎 |  | 🞎 | 🞎 |
|  | 🞎 | 🞎 |  | 🞎 | 🞎 |
|  | 🞎 | 🞎 |  | 🞎 | 🞎 |
|  | 🞎 | 🞎 |  | 🞎 | 🞎 |
|  | 🞎 | 🞎 |  | 🞎 | 🞎 |
|  | 🞎 | 🞎 |  | 🞎 | 🞎 |
|  | 🞎 | 🞎 |  | 🞎 | 🞎 |
|  | 🞎 | 🞎 |  | 🞎 | 🞎 |
|  | 🞎 | 🞎 |  | 🞎 | 🞎 |
|  | 🞎 | 🞎 |  | 🞎 | 🞎 |
|  | 🞎 | 🞎 |  | 🞎 | 🞎 |
|  | 🞎 | 🞎 |  | 🞎 | 🞎 |
|  | 🞎 | 🞎 |  | 🞎 | 🞎 |
|  | 🞎 | 🞎 |  | 🞎 | 🞎 |
|  | 🞎 | 🞎 |  | 🞎 | 🞎 |
|  | 🞎 | 🞎 |  | 🞎 | 🞎 |
|  | 🞎 | 🞎 |  | 🞎 | 🞎 |
|  | 🞎 | 🞎 |  | 🞎 | 🞎 |
|  | 🞎 | 🞎 |  | 🞎 | 🞎 |
|  | 🞎 | 🞎 |  | 🞎 | 🞎 |
|  | 🞎 | 🞎 |  | 🞎 | 🞎 |
|  | 🞎 | 🞎 |  | 🞎 | 🞎 |

**Work instructions for team “Dismemberment”**


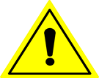

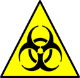

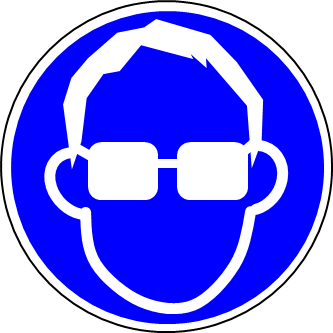

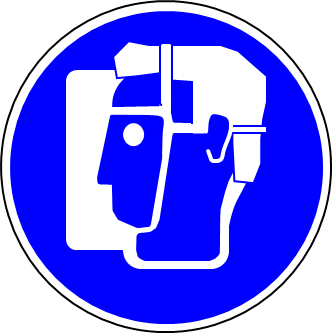

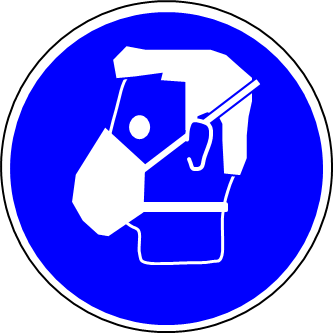

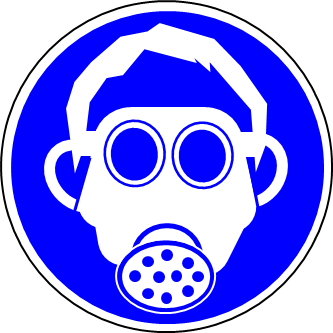

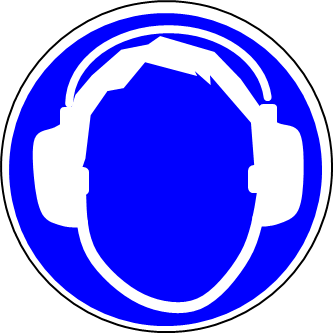

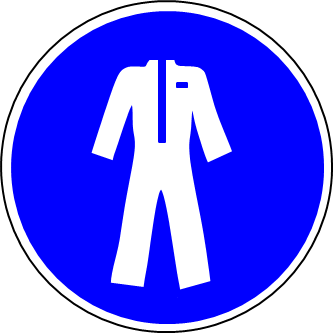

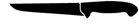

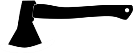

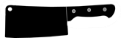

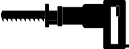

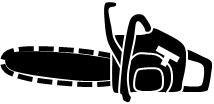


1. Unload and **place elephant body in lateral recumbency** (healthy side down).
2. **Fix, secure & lift head**. **Mobilize** **tongue, pharynx, larynx (I.), and neck organs** (trachea, adjacent soft tissues, and organs (vessels, lymph nodes, para/thyroid glands, etc.) **and leave connected to the lungs**. Examine lymph nodes (team “Locomotion”).

**I.
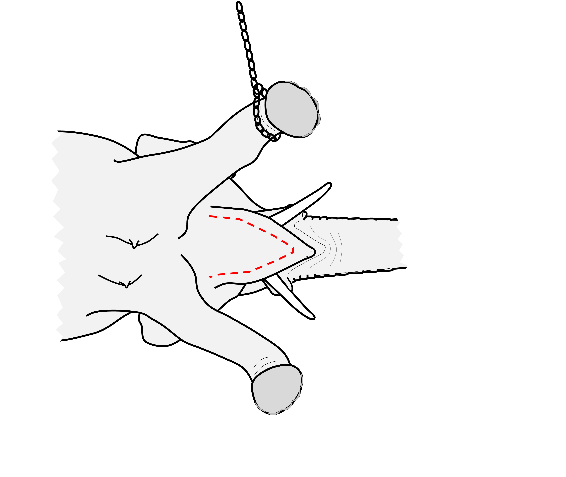
II.
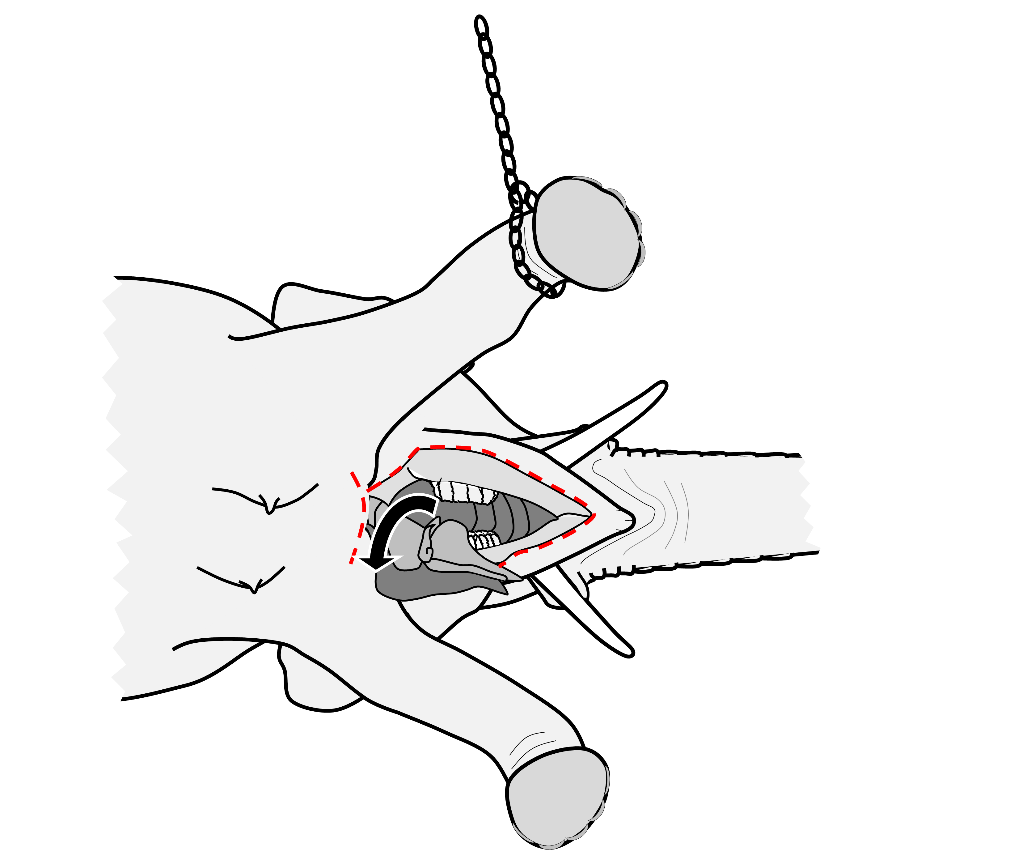
 II.
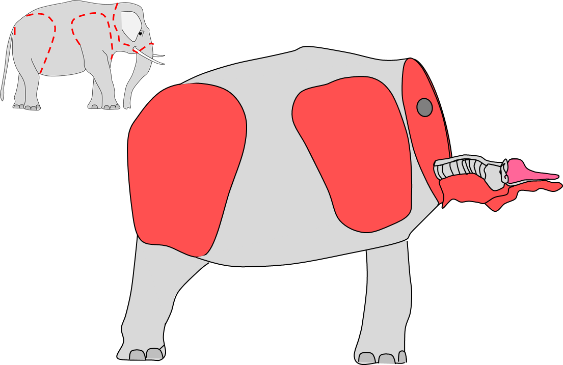
**

**I, II**. Ventral aspect. The red dotted line indicates the cutting line for removal of the tongue and neck organs. Cut through the chin/neck skin into the oral cavity, following the medial contours of the lower jaw to the chin. **II.** Remove limbs and head.

1. **Disarticulate the head** in the atlanto-occipital joint, **remove head** & pass to team “Head” (**II, III**).
2. **Fix, secure, lift and remove upper front foot** & hand to team “Locomotion” (if not identical with team “Dismemberment”) (**II, III**).
3. **Fix, secure, lift and remove upper front leg with shoulder** & hand to team “Locomotion” (**II, III**).
4. **Remove mammary gland** & hand to team UGT.
5. **Fix, secure, lift and remove upper hind foot** & hand to team “Locomotion” (**II, III**).
6. **Fix, secure, lift and separate upper hind leg in coxo-femoral joint, remove leg** & hand to team “Locomotion” (**II, III**).

**III.
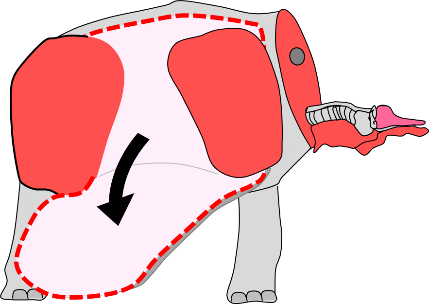
IV.
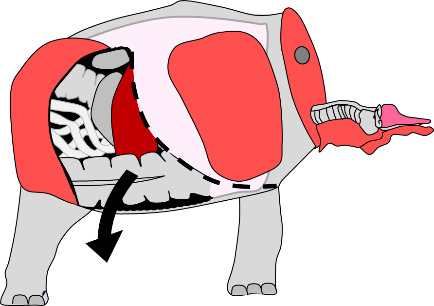
V.**
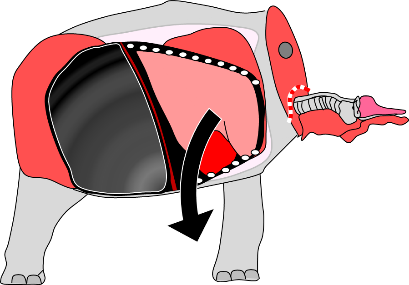


**III**. Remove skin from lateral body wall. I**V.** Remove lateral abdominal body wall, eviscerate abdominal & pelvic cavy. **V.** Remove lateral thoracic wall and eviscerate thoracic organs.

1. **Remove skin from upper lateral abdomen and thorax** (**IV**).
2. In male elephants, **remove prepuce and penis** & hand to team “UGT”.
3. **Remove abdominal wall** (**V**). Examine abdominal and pelvic cavity (sampling: Team “GIT”)
4. **Remove spleen, small and large intestines, stomach, mesentery and omentum, pancreas,** and **liver** & hand to team “GIT”. **Remove abdominal aorta** & hand over to team “Thorax”.
5. **Remove kidneys, urinary bladder, reproductive organs and adrenal glands** & hand to team “UGT”.
6. **Remove lateral thoracic wall** (**VI**). Mobilize lungs from thoracic walls & examine thorax.
7. **Remove lungs, heart, thymus, mediastinum, and neck organs** & pass the entire pluck to team “Thorax”.
8. Proceed with dissection of the head, supporting team “Head”.
9. **Fix, secure, lift and turn carcass, remove the contralateral limbs/feet**.
10. Support team “Locomotion” with **dissection of joints**, **musculature**, **peripheral nerves** and **feet**,

and **vertebral column** **and removal of spinal cord** (if required).

19. Further **dismember carcass for disposal**.

**Work instructions for team “Head”**


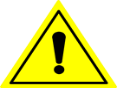

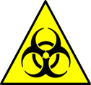

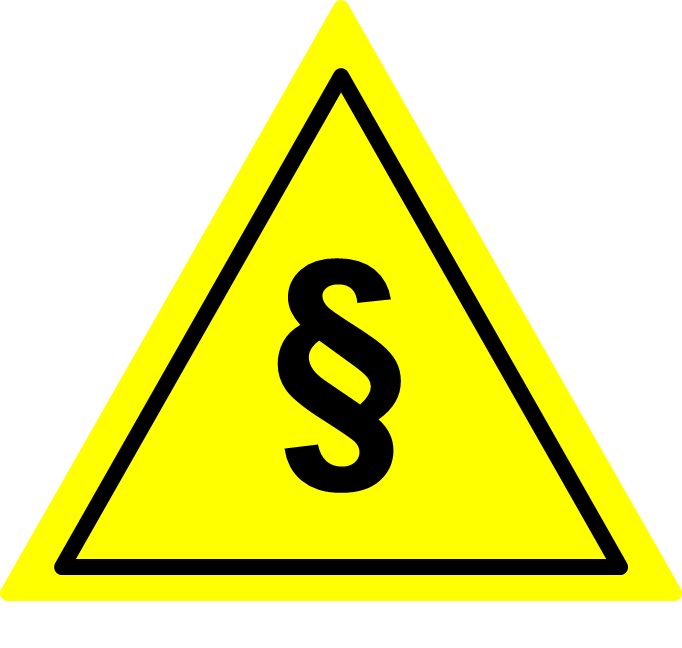

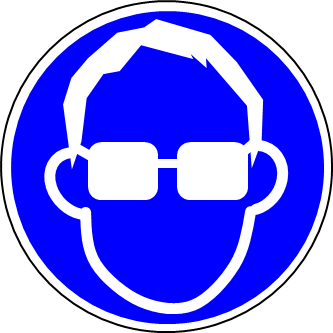

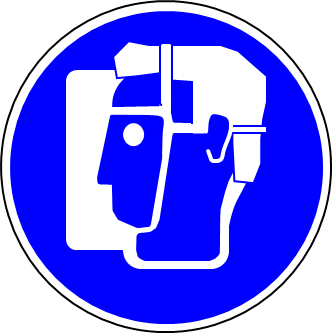

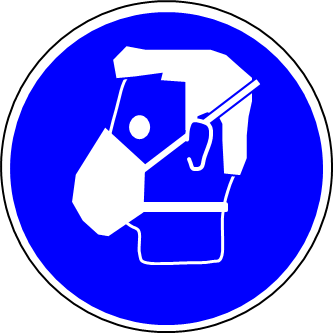

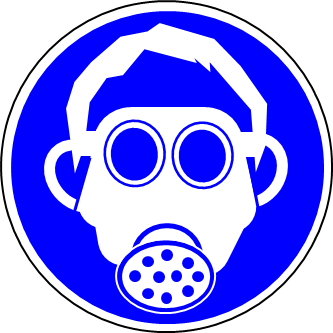

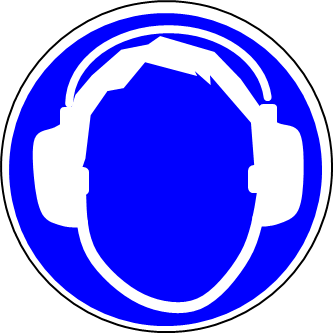

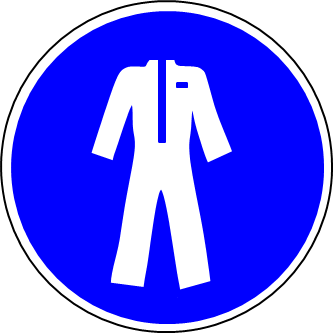


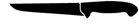

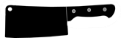

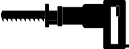

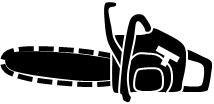

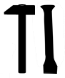

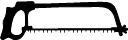


1. **Remove trunk at its base** and hand to team “Thorax”.
2. **Remove and examine eyes** and Harderian glands.
3. Examine temporal glands.
4. Turn head (ventral side facing up), fix lower jaw at the chin on the crane hook and lift until the jaw opens. **Disarticulate and examine temporomandibular joints and separate lower jaw from head**.
5. Turn head (ventral side facing down). **Remove ears and skin** from the skullcap. **Access the brain dorsally** (**I-III**).


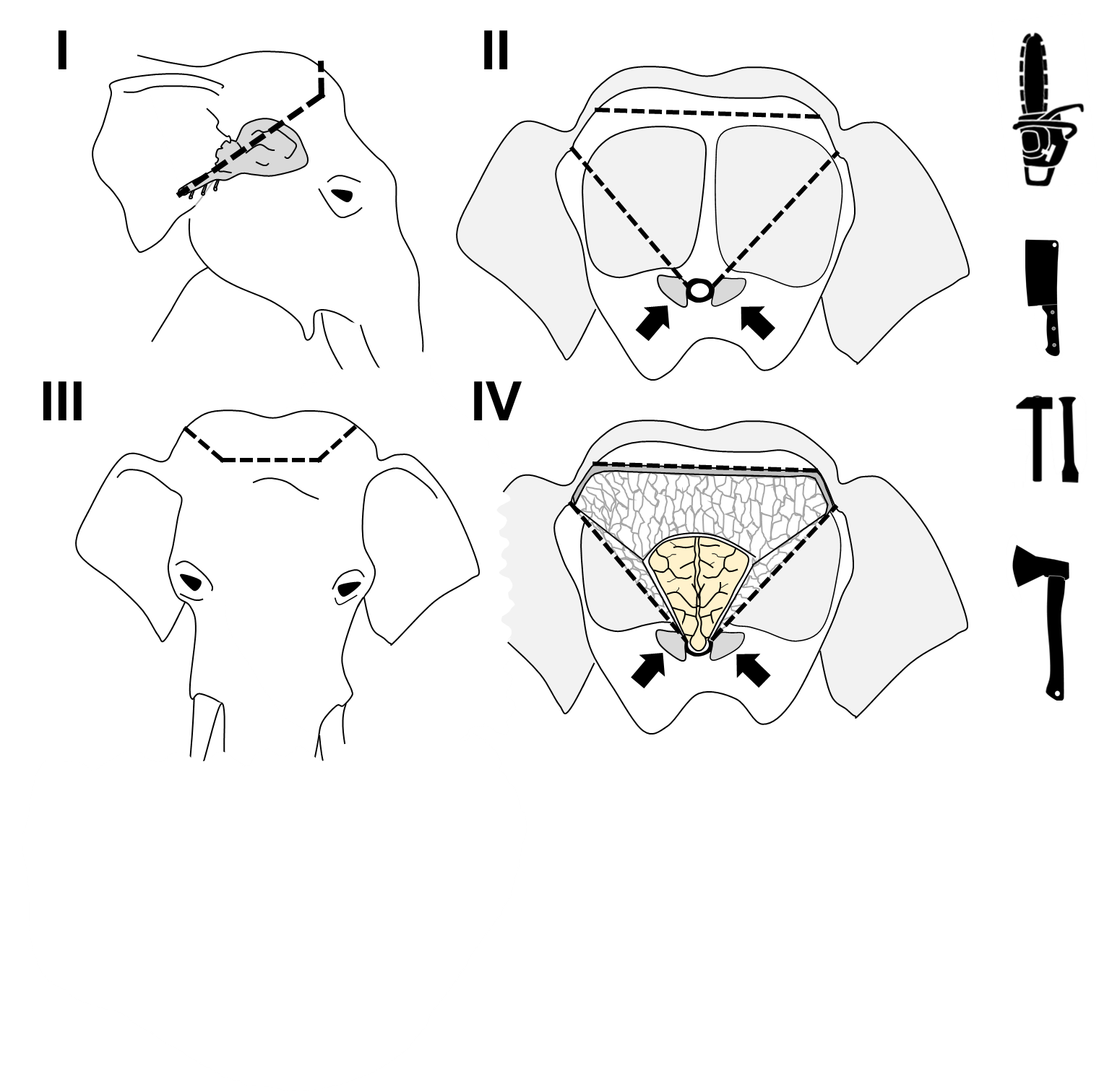


**I-III**. Removal of the brain. The red-dotted lines indicate incision lines. **I.** Lateral aspect. The position of the brain is indicated. **II.** Caudal aspect. Incision lines are elongated into the foramen magnum, dorsal of the condyles. **III.** Frontal aspect. **IV.** Location of the brain after removal of the skullcap and the extensive sinus. Pay attention to formation of sharp bone splinters when working through the skullcap (wear face shield/eye protection). Note that unlike in the presented sketch, the pinnae, the lower jaw and the skin are removed from the head prior to dissection of the brain.

1. **Examine and remove brain and meninges**.
2. Locate, examine and remove **trigeminal ganglia**, **optical nerves**, and **pituitary gland**.
3. **Dissect/open the external ear canal**.
4. **Examine teeth**, **remove and examine tusks**. Keep tusks in a safe place.
5. Locate and examine the vomeronasal (Jacobson’s) organ.
6. **Dissect/open and examine paranasal sinuses and dental alveoli.**
7. Split head sagittally and dissect inner ear(s) (optional).
8. Process skull/jaws for decocting (80°C, 12-24 hrs) and remove teeth after decocting (optional).

**Sampling instructions for team “Head”**

- **Pathological findings/alterations**

Upon identification of lesions **call the pathologist in charge**/superintendent to inspect the lesion and decide what samples should be taken. Common lesions of the head include **sinusitis** and **fractured teeth**.

- Call the **photographer** to document the lesion;
- Call a member of the **“Sampling” team** to t**ake specimens for subsequent microbiological (bacteriology, virology, mycology) or parasitological analyses with sterile instruments or swabs** before excision of tissue samples;
- **Excise the lesion spaciously** **and place the excised sample in/on a suitable vessel** (*e.g.,* disposable paper plates/bowels). **Label the sample** appropriately (tissue type, location) and **indicate the sample types to be collected and their numbers**, if applicable [*e.g.,* FFPE-histology, transmission electron microscopy (3x), samples for molecular analyses (2x)], and **hand over to the “Sampling” team**, that will further process the sample(s).
- **Standardly scheduled organ/tissue samples**

**The following specific locations of tissues/organs are sampled**. Sample sizes are specified below. **Excise samples and place them on a** **disposable paper plate**/**bowel**. **Label the sample** appropriately (tissue type, location) and **hand over to the “Sampling” team** that will further process the sample(s).

| **Team “Head”** | | | |
| --- | --- | --- | --- |
| **Necropsy number:** | | **Date:** | |
| **Organ** | **Location/direction** | **Size/volume** | **N° of samples** |
| **Eyes** | Remove complete eye bulbs. Indicate left and right eye. |  | 2 |
| **Brain and meninges** | Remove completely. |  | 1 |
| **Pituitary gland** | Remove completely. |  | 1 |
| **Trigeminal ganglion** | Excise both. Indicate left and right side. |  | 2 |
| **Tusks**  **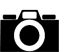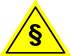** | Extract both tusks (if present). | Weight (kg)  left: __________  right: __________  🞎 fresh 🞎 cooked, dry | (*store safely until retrieval by authorized persons or disposal)* |
|  |  |  |  |
|  |  |  |  |
|  |  |  |  |
|  |  |  |  |
|  |  |  |  |

**Work instructions for team “Locomotion”**


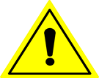

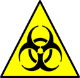

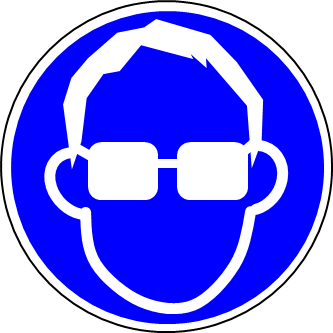

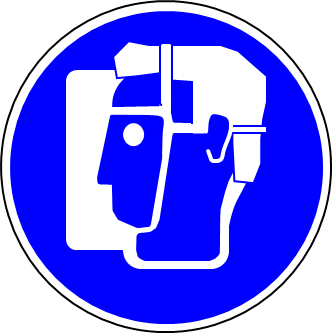

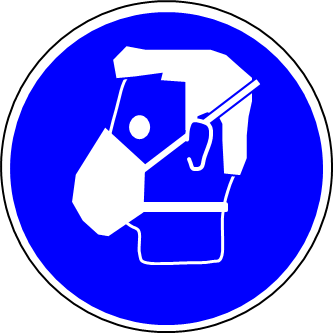

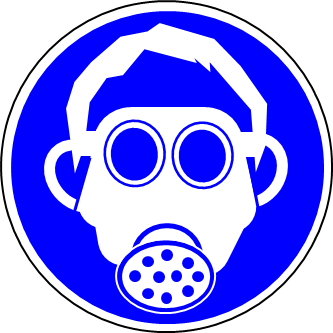

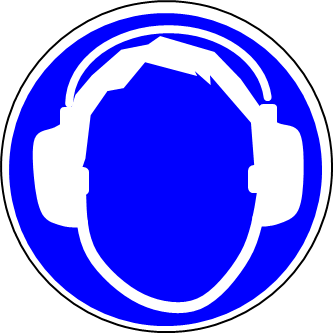

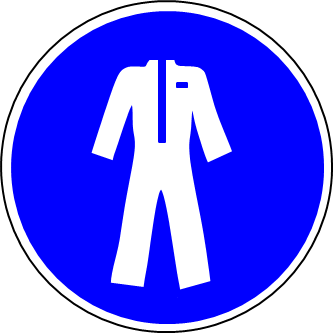

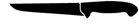

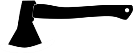

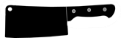

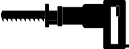

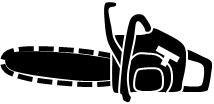


The **“Locomotion” team** is responsible for the external examination as well as the examination and sampling of **skin, peripheral lymph nodes, skeletal muscles, joints, and skeleton** (limbs, feet, vertebral column, spinal cord). This may include **application of imaging techniques such as X-ray, CT, MRI** (*e.g.,* of feet) as scheduled in advance of the necropsy. Moving, dissecting, and sawing of heavy animal parts constitutes a considerable risk. **Always strictly adhere to the given workplace safety and infection protection rules**.

Perform **External Examination**:

1. Document any additional identifying markers (transponders, tattoos, distinguishing features)
2. If not determined by scale, estimate the elephant body weight (W) according to Sreekumar and Nirmalan (1989) from the body length L (base of the forehead to the base of the tail), and the chest girth G, as follows: **W [kg]=−1010+0.036*(L [cm]×G [cm])**.
3. Examine skin, ears, body orifices, prepuce, and mammary gland. If appropriate, order sterile collection of samples/swabs by team “Sampling” and photo documentation. Take tissue samples as necessary/scheduled.

During dismemberment of the elephant body (by team “Dismemberment):

1. Carefully locate, examine and section peripheral lymph nodes. Start with the superficial cervical and mandibular lymph nodes, subsequent to mobilization of the tongue, pharynx, larynx, trachea, esophagus and adjacent tissues (team “Dismemberment”). If appropriate, order photo documentation. Sample lymph nodes (peripheral lymph nodes of the neck are sampled by team “Thorax”). Label lymph node samples appropriately and pass to the “Sampling” team. If lymph nodes display signs suspicious of TB infection (granulomatous/caseous lymphadenitis, calcification) immediately inform the pathologist in charge (switch to higher safety classes of respiratory protection, if appropriate). Examine further popliteal, spf. inguinal, & peripheral axillar lymph nodes.
2. Label feet and limbs appropriately (right/left; front/back) before proceeding with the dissection.
3. Dissect, examine, and sample (if scheduled/appropriate) acetabulofemoral, knee, shoulder, elbow, tarsal, and carpal joints.
4. Dissect, examine and sample (if scheduled/appropriate) skeletal muscles, tendons, tendon sheaths, synovial bursa sacs, large peripheral nerves, and vessels of limbs.
5. Examine and sample (if scheduled/appropriate) the musculature of the back.
6. Examine the feet. For detection of bone lesions, imaging technologies such as CT can be helpful. Dissect feet (according to CT findings) by sawing to access phalangeal and sesamoid bones and joints and the large “cushion pad” of connective tissue above the sole. Ask the “photographer” to take images of gross findings and collect samples, if scheduled/appropriate.
7. Upon completion of the examination/sampling process, wait for approval by the pathologist in charge before disposing of the body parts.

**Sampling instructions for team “Locomotion”**

- **Pathological findings/alterations**

Upon identification of lesions **call the pathologist in charge**/superintendent to inspect the lesion and decide what samples should be taken.

- Call the **photographer** to document the lesion.
- Call a member of the **“Sampling” team** to t**ake specimens for subsequent microbiological (bacteriology, virology, mycology) or parasitological analyses with sterile instruments or swabs** before excision of tissue samples.
- **Excise the lesion spaciously** **and place the excised sample in/on a suitable vessel** (*e.g.,* disposable paper plates/bowels). **Label the sample** appropriately (tissue type, location) and **indicate the sample types to be collected and their numbers**, if applicable [*e.g.,* FFPE-histology, transmission electron microscopy (3x), samples for molecular analyses (2x)], and **hand over to the “Sampling” team** that will further process the sample(s).
- **Standardly scheduled organ/tissue samples**

**The following specific locations of tissues/organs are sampled**. Sample sizes are specified below. **Excise samples and place them on a** **disposable paper plate**/**bowel**. **Label the sample** appropriately (tissue type, location) and **hand it over to the “Sampling” team** that will further process the sample(s).

| **Team “Locomotion”** | | | |
| --- | --- | --- | --- |
| **Necropsy number:** | | **Date:** | |
| **Organ** | **Location/direction** | **Size/volume** | **N° of samples** |
| **Lymph node:** |  |  |  |
| **Lymph node:** |  |  |  |
| **Lymph node:** |  |  |  |
| **Lymph node:** |  |  |  |
| **Lymph node:** |  |  |  |
|  |  |  |  |
|  |  |  |  |
|  |  |  |  |
|  |  |  |  |
|  |  |  |  |
|  |  |  |  |
|  |  |  |  |
|  |  |  |  |
|  |  |  |  |
|  |  |  |  |

**Work instructions for team “Gastrointestinal System” [GIT]**


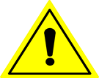

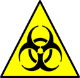

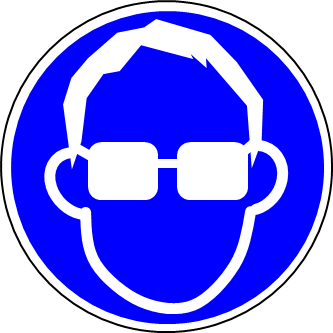

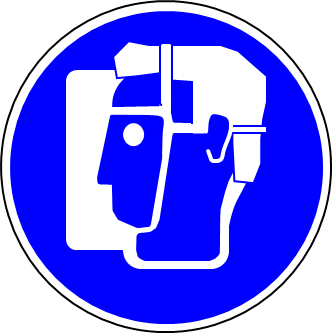

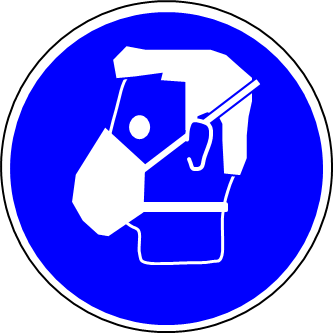


1. Upon opening of the abdominal cavity and removal of the lateral abdominal wall (by team “Dismemberment”), **examine the abdominal cavity** (peritoneum, effusions, position of organs) and, if appropriate, order sterile collection of tissue/effusion/swab-samples by team “Sampling” and photo documentation of findings.
2. After evisceration, shortly view organs in place, then **drag organs away from the dismemberment-place to the allocated place for team “GIT”**, to allow the progress of the dismemberment process. Due to the heavy weight, examination of intestines is usually performed on the ground, while spleen, liver, and stomach can be placed on necropsy tables for better access.
3. **Examine and dissect the spleen**. To examine the spleen parenchyma, cut serially through the entire organ (make incomplete sections, so the spleen will hold together). Take samples as appropriate/scheduled.
4. **Examine and dissect the liver**. To examine the liver parenchyma, cut serially through the entire organ (make incomplete sections, so the liver will hold together). Take samples as appropriate/scheduled.
5. Spread the intestines on the ground. **Locate and examine mesenteric lymph nodes**. Take and label lymph node samples appropriately, as scheduled and pass to the “Sampling” team. **If lymph nodes display signs suspicious of TB infection (granulomatous/caseous lymphadenitis, calcification) immediately inform the pathologist in charge** (switch to higher safety classes of respiratory protection, if appropriate).
6. **Locate, examine, and remove the pancreas.** Take samples as appropriate/scheduled.
7. Remove the stomach, open it along the great curvature, remove and weigh the stomach contents (take samples of stomach content, if appropriate). **Examine the stomach** mucosa. Take samples as appropriate/scheduled.
8. Remove the mesentery from the intestines. Take samples as appropriate/scheduled.
9. **Display intestines** (duodenum, jejunum, ileum, caecum, colon, rectum) longitudinally on the ground. Measure the lengths of intestinal segments, if appropriate.
10. Open intestines longitudinally and **examine intestinal contents** (take samples of ingesta/feces, if appropriate).
11. **Remove intestinal contents/feces** (this is usually extensive work; elephant intestines may contain several wheelbarrow-loads of ingesta).
12. **Examine the intestinal mucosae**. Take organ/tissue samples as appropriate/scheduled.
13. Get the esophagus (cervical part and thoracic part) from team “Thorax”.
14. **Examine the esophagus**. Take samples as appropriate/scheduled.
15. **After the necropsy, leave organs/tissues and removed ingesta in place - do not dispose of any tissues before approval by the pathologist in charge.**

**Sampling instructions for team “Gastrointestinal System” [GIT]**

- **Pathological findings/alterations**

Upon identification of lesions **call the pathologist in charge**/superintendent to inspect the lesion and decide what samples should be taken.

- Call the **photographer** to document the lesion.
- Call a member of the **“Sampling” team** to t**ake specimens for subsequent microbiological (bacteriology, virology, mycology) or parasitological analyses with sterile instruments or swabs** before excision of tissue samples.
- **Excise the lesion spaciously** **and place the excised sample in/on a suitable vessel** (*e.g.,* disposable paper plates/bowels). **Label the sample** appropriately (tissue type, location) and **indicate the sample types to be collected and their numbers**, if applicable [*e.g.,* FFPE-histology, transmission electron microscopy (3x), samples for molecular analyses (2x)], and **hand over to the “Sampling” team** that will further process the sample(s).
- **Standardly scheduled organ/tissue samples**

**The following specific locations of tissues/organs are sampled**. Sample sizes are specified below. **Excise samples and place them on a** **disposable paper plate**/**bowel**. **Label the sample** appropriately (tissue type, location) and **hand over to the “Sampling” team** that will further process the sample(s).

| **Team “Gastrointestinal System” [GIT]** | | | |
| --- | --- | --- | --- |
| **Necropsy number:** | | **Date:** | |
| **Organ** | **Location/direction** | **Size/volume** | **N° of samples** |
| **Lymph node:** |  |  |  |
| **Lymph node:** |  |  |  |
| **Lymph node:** |  |  |  |
| **Spleen** |  |  |  |
| **Liver** |  |  |  |
| **Pancreas** |  |  |  |
| **Mesentery** |  |  |  |
| **Esophagus** |  |  |  |
| **Stomach** |  |  |  |
| **Duodenum** |  |  |  |
| **Jejunum** |  |  |  |
| **Ileum** |  |  |  |
| **Cecum** |  |  |  |
| **Colon** |  |  |  |
| **Rectum** |  |  |  |

**Work instructions for team “Urogenital system” [UGT]**

1. After evisceration of the abdominal organs of the gastrointestinal system and the spleen, **locate and shortly view organs in place** (kidneys, ureter/testes & epididymides, urinary bladder, adrenals and uterus). If visible, note lesions, and arrange for photo documentation, if appropriate.
2. After removal of the urogenital system organs (by team “Dismemberment”), **transport (drag, if uterus is pregnant) organs away from the dismemberment-place to the allocated place for team “UGT”,** to allow the progress of the dismemberment process. Due to the heavy weight, examination of pregnant uteri in advanced gestation stages is usually performed on the ground, while the other UGT-organs can be placed on necropsy tables for better access.
3. **Locate and examine profound inguinal lymph nodes** (either adjacent to the eviscerated UGT-organs or remained in the carcass). Take and label lymph node samples appropriately, as scheduled and pass to the “Sampling” team. **If lymph nodes display signs suspicious of TB infection (granulomatous/caseous lymphadenitis, calcification) immediately inform the pathologist in charge** (switch to higher safety classes of respiratory protection, if appropriate).
4. **Examine and dissect the kidneys**. Perform several sections through the parenchyma to examine the organ. Take samples as necessary/scheduled and label right/left kidney samples appropriately.
5. **Examine and dissect the ureters**. Take samples as necessary/scheduled.
6. **Examine and dissect the urinary bladder**. Take samples as necessary/scheduled.
7. If the **abdominal aorta** is present adjacent to the eviscerated UGT-organs, examine from outside for vascular anomalies (*e.g.,* aneurysms - if present, arrange photo documentation and inform team “Thorax”). Remove abdominal aorta (including adjacent arteries, and caval vein) and **hand to team “Thorax”.**
8. **In male elephants**, l**ocate, remove, and examine testes, epididymides, and the spermatic duct/vas deferens**. Take samples as necessary/scheduled and label right/left sides appropriately.

**-Examine and dissect the urethra, prostate, seminal vesicles, bulbourethral gland, penis, and prepuce** (penis and prepuce are handed over by team “Dismemberment”). Take samples as necessary/scheduled.

1. **In female elephants**, **examine and dissect the ovaries and Fallopian tubes**. **Watch out for ovary cysts, follicles/corpora lutea, and tumors.** Take samples as necessary/scheduled and label right/left sides appropriately.

**-Examine and dissect the uterus**. Open uterus longitudinally. **Watch out for endometrial cysts and tumors (frequent)**. Take samples as necessary/scheduled. **If pregnant, examine the fetus (note size, weight, position, perform complete necropsy), placenta/embryonic membranes, and umbilical cord.** Take samples as necessary/scheduled.

-**Examine and dissect** **cervix and vagina, vulva, and clitoris**. Take samples as necessary/scheduled.

1. **Locate and examine the adrenal glands**. Perform several sections through the parenchyma to examine the organs. Take samples as necessary/scheduled and label right/left adrenal gland samples appropriately.
2. Check if the **mammary gland** was appropriately examined by team “Locomotion”.
3. **After the necropsy, leave organs/tissues in place - do not dispose of any tissues, before approval by the pathologist in charge.**

**Sampling instructions for team “Urogenital System” [UGT]**

- **Pathological findings/alterations**

Upon identification of lesions **call the pathologist in charge**/superintendent to inspect the lesion and decide what samples should be taken.

- Call the **photographer** to document the lesion.
- Call a member of the **“Sampling” team** to t**ake specimens for subsequent microbiological (bacteriology, virology, mycology) or parasitological analyses with sterile instruments or swabs** before excision of tissue samples.
- **Excise the lesion spaciously** **and place the excised sample in/on a suitable vessel** (*e.g.,* disposable paper plates/bowels). **Label the sample** appropriately (tissue type, location) and **indicate the sample types to be collected and their numbers**, if applicable [*e.g.,* FFPE-histology, transmission electron microscopy (3x), samples for molecular analyses (2x)], and **hand over to the “Sampling” team**, that will further process the sample(s).
- **Standardly scheduled organ/tissue samples**

**The following specific locations of tissues/organs are sampled**. Sample sizes are specified below. **Excise samples and place them on a** **disposable paper plate**/**bowel**. **Label the sample** appropriately (tissue type, location) and **hand over to the “Sampling” team**, who will further process the sample(s).

| **Team “Urogenital System” [UGT]** | | | |
| --- | --- | --- | --- |
| **Necropsy number:** | | **Date:** | |
| **Organ** | **Location/direction**  (indicate left and right) | **Size/volume** | **N° of samples** |
| **Lymph node:** |  |  |  |
| **Lymph node:** |  |  |  |
| **Lymph node:** |  |  |  |
| **Kidneys** |  |  |  |
| **Adrenal glands** |  |  |  |
| **Urinary bladder** |  |  |  |
| **Testis, epididymis** |  |  |  |
| **Ovary** |  |  |  |
| **Uterus** |  |  |  |
|  |  |  |  |
|  |  |  |  |
|  |  |  |  |
|  |  |  |  |

**Work instructions for team “Thorax”**

1. **Thoracic and neck organs (pluck) are** provided by team “Dismemberment”. Start with the neck organs:

- **If not already done by team “Locomotion”, locate and examine mandibular and retropharyngeal lymph nodes**. Take and label lymph node samples appropriately, as scheduled and pass to the “Sampling”-team. **If lymph nodes display signs suspicious of TB infection (granulomatous/caseous lymphadenitis, calcification) immediately inform the pathologist in charge** (switch to higher safety classes of respiratory protection, if appropriate).

-Briefly examine and **remove tongue and esophagus & hand to team “GIT”**.

-**Dissect and examine thyroid and parathyroid glands**.

-**Dissect and examine cervical vessels and nerves**.

-**Dissect and examine pharynx, larynx, and the cervical part of the trachea**.

Order photo documentation and take samples as appropriate/scheduled.

1. Obtain great abdominal vessels (aorta, caval vein) from team “Dismemberment”, or team “UGT”. **Dissect and examine great abdominal vessels** (**watch out for aortic aneurysms**), order photo documentation and take samples as appropriate/scheduled.
2. Upon opening of the thorax and removal of the lateral thoracic wall (team “Dismemberment”) and mobilization of the lungs (disconnect the parietal pleura from the thoracic wall), **examine the organs inside the thorax** (pleura, position of organs) and, if appropriate, order sterile collection of tissue /swab-samples by team “Sampling” and photo documentation of findings. **If lymph nodes/lung display signs suspicious of TB infection (granulomatous/caseous inflammation, calcification) immediately inform the pathologist in charge** (switch to higher safety classes of respiratory protection, if appropriate).
3. After evisceration of thoracic organs, shortly examine organs in place, **then move organs away from the dismemberment-place to the allocated place for team “Thorax”**, to allow the progress of the dismemberment process.
4. **Examine and dissect the mediastinum, remaining lymph nodes, trachea, and bronchi**. If appropriate, order sterile collection of tissue/swab-samples by team “Sampling” and photo documentation of findings. **If lymph nodes/lung display signs suspicious of TB infection (granulomatous/caseous inflammation, calcification) immediately inform the pathologist in charge.**
5. Locate and **remove the esophagus and hand over to team “GIT”.**
6. **Locate and examine the thymus**. Take samples as appropriate/scheduled.
7. Remove lungs from the heart and adjacent pericardium/great vessels. **Dissect and examine lungs** thoroughly - cut serially through the entire organ (make incomplete sections, so the lung parenchyma will hold together). Take samples as appropriate/scheduled.
8. **Dissect and examine the pericardium**. Take samples as appropriate/scheduled.
9. **Dissect and examine the heart** (note the incised heart apex – normal finding in elephants): Examine the **epicardium**, dissect right and left **atria and ventricles**, **vascular trunks**. Examine **endocardium**, **heart valves** and **coronary vessels**. Examine **myocardium** – place serial sections through the myocardium). Dissect and examine **aorta**, **caval vein**, **pulmonary artery,** and **veins**. Take samples as appropriate/scheduled.
10. **Obtain the trunk from team “Head”**. **Dissect and examine the trunk**: Completely open both nostrils/nasal passages longitudinally. Take samples as appropriate/scheduled.
11. **After the necropsy, leave organs/tissues and removed ingesta in place - do not dispose of any tissues before approval by the pathologist in charge.**

**Sampling instructions for team “Thorax”**

- **Pathological findings/alterations**

Upon identification of lesions **call the pathologist in charge**/superintendent to inspect the lesion and decide what samples should be taken.

- Call the **photographer** to document the lesion.
- Call a member of the **“Sampling” team** to t**ake specimens for subsequent microbiological (bacteriology, virology, mycology) or parasitological analyses with sterile instruments or swabs** before excision of tissue samples.
- **Excise the lesion spaciously** **and place the excised sample in/on a suitable vessel** (*e.g.,* disposable paper plates/bowels). **Label the sample** appropriately (tissue type, location) and **indicate the sample types to be collected and their numbers**, if applicable [*e.g.,* FFPE-histology, transmission electron microscopy (3x), samples for molecular analyses (2x)], and **hand over to the “Sampling” team**, that will further process the sample(s).
- **Standardly scheduled organ/tissue samples**

**The following specific locations of tissues/organs are sampled**. Sample sizes are specified below. **Excise samples and place them on a** **disposable paper plate**/**bowel**. **Label the sample** appropriately (tissue type, location) and **hand over to the “Sampling” team** that will further process the sample(s).

| **Team “Thorax”** | | | |
| --- | --- | --- | --- |
| **Necropsy number:** | | **Date:** | |
| **Organ** | **Location/direction** | **Size/volume** | **N° of samples** |
| **Lymph node:** |  |  |  |
| **Lymph node:** |  |  |  |
| **Lymph node:** |  |  |  |
| **Lymph node:** |  |  |  |
| **Lymph node:** |  |  |  |
| **Lung** |  |  |  |
| **Heart** |  |  |  |
|  |  |  |  |
|  |  |  |  |
|  |  |  |  |
|  |  |  |  |
