# Supplementary material for: A pictural guide to postmortem examination of elephants
Source: PLoS One. 2026 Feb 9;21(2):e0338783. doi: 10.1371/journal.pone.0338783 (PMC12885571; doi:10.1371/journal.pone.0338783)
Supplement: S3 File — LMU-guide to systematic dissection of an elephant brain including table of S2 Video sections. (DOCX) [file pone.0338783.s004.docx]

**S3 Material. Dissection of an Elephant Brain**

**LMU-Guide to Systematic Dissection of an Elephant Brain**

The brain trimming protocol introduced herein bases on the experiences collected during post-mortem examinations of 19 elephants dissected at the Institute of Veterinary Pathology at the LMU Munich and follows the algorithms posted for other species [1, 2]. Details on gross anatomy are available in several comprehensive articles published by experienced researchers [3-6]. Apart from the sheer volume of the elephant brain, challenges are in particular the complex three-dimensional architecture of the cerebellum with its tonsil-like paravermal parts and the highly variable anatomy of the rather small and asymmetric hippocampi [3, 6]. As in people, the caudal (posterior) part of the hippocampi does not flex dorsorostrally to liaise in a hippocampal midline commissure, as is the case in most quadruped mammals [3]. This part only comprises a thin tissue band that grossly often missed the seahorse pattern, with discernible dentate gyrus and cornu ammonis, that lead to its name. It is therefore easy to miss during dissection and sampling. The thick rostral (anterior) temporoventral part of the hippocampus, on the other hand, is highly convoluted so that it is quite difficult to create proper perpendicular sections that are required to gain insight into hippocampal segment-specific pathologies. The "shell-out" technique introduced in the video resembles the most promising method to achieve perpendicular sections after gross prelocalisation of cornu ammonis.

The video contains the following sections:

| Section |  |  | Time (min:sec) |
| --- | --- | --- | --- |
| Anatomic overview | Dorsal view |  | 00:04-00:14 |
|  | Lateral view |  | 00:18-00:23 |
|  | Caudal view |  | 00:26-00:34 |
|  | Ventral view |  | 00:37-00:44 |
| Hindbrain dissection | Separation of hindbrain |  | 00:51-10:07 |
|  | Cerebellar & pons dissection | Midcerebellum | 01:26-02:21 |
|  |  | Sagittal Vermis | 02.22-02:48 |
|  |  | Rostroventral hemispheres & pons | 02:50-03:59 |
|  |  | Rostral paravermis | 04:03-04-33 |
|  |  | Outer hemisphere of rostral lobe | 04:34-05:09 |
|  |  | Caudal vermis and paravermis | 05:43-06:09 |
|  |  | Dorsal outer hemisphere of caudal lobe | 06:11-06:30 |
|  |  | Caudoventral hemispheres | 06:31-06:45 |
|  | Lower brainstem |  | 05:10-05:42 |
|  | Overview of hindbrain slabs |  | 06.46-07:05 |
| Frontal lobe dissection | Technique |  | 07:07-08:08 |
|  | Overview of slabs |  | 08:09-08:12 |
| Dissection of hippocampus, parietal and occipital lobes, thalamus and mesencephalon | Dorsal & occipital part of hippocampus dissection |  | 08:13-09:46  11:05-11:24 |
|  | Parietal lobe dissection |  | 09:48-09:58 |
|  | Occipital lobe dissection |  | 09:58-10:43  13:17-13:47 |
|  | Mesencephalon and thalamus dissection |  | 08:15-08:40  10:44-11:04 |
|  | Temporoventral part of hippocampus dissection | Transverse sections | 11:24-12:00 |
|  |  | “Shell-out” technique | 12:02-13:16 |
| Overview of forebrain and midbrain slabs |  |  | 13:17-14:01 |
| Overview of all brain slabs |  |  | 14:02-14:09 |

1. Bitschi ML, Bago Z, Rosati M, Reese S, Goehring LS, Matiasek K. A systematic approach to dissection of the equine brain—evaluation of a species-adapted protocol for beginners and experts. Front Neuroanat. 2020;14:614929.
2. Matiasek K, Pumarola IBM, Rosati M, Fernandez-Flores F, Fischer A, Wagner E, et al. International Veterinary Epilepsy Task Force recommendations for systematic sampling and processing of brains from epileptic dogs and cats. BMC Vet Res. 2015;11:216.
3. Hakeem AY, Hof PR, Sherwood CC, Switzer RC 3rd, Rasmussen LE, Allman JM. Brain of the African elephant (Loxodonta africana): neuroanatomy from magnetic resonance images. Anat Rec A Discov Mol Cell Evol Biol. 2005;287:1117–1127.
4. Herculano-Houzel S. Mammalian neurobiology: the elephant (brain) in the room. Curr Biol. 2022;32:R176–R178.
5. Herculano-Houzel S, Avelino-de-Souza K, Neves K, Porfirio J, Messeder D, Mattos Feijo L, et al. The elephant brain in numbers. Front Neuroanat. 2014;8:46.
6. Shoshani J, Kupsky WJ, Marchant GH. Elephant brain. Part I: gross morphology, functions, comparative anatomy, and evolution. Brain Res Bull. 2006;70:124–157.
