# Supplementary material for: A pictural guide to postmortem examination of elephants
Source: PLoS One. 2026 Feb 9;21(2):e0338783. doi: 10.1371/journal.pone.0338783 (PMC12885571; doi:10.1371/journal.pone.0338783)
Supplement: S1 Table — (DOCX) [file pone.0338783.s005.docx]

**S1 Table. Materials and equipment checklist.**

| **Materials** | | **Amount** | **Tick box** | **Notes** |
| --- | --- | --- | --- | --- |
| **Personnel protective equipment (PPE)** | - First aid kit - Disposable waterproof coveralls - Rubber boots with slip-resistant soles - Disposable gloves - Disposable caps/hoods - Protective goggles and face shields - Duct tape | 2/10 pers.*  2 pp**  1 pp  stock up  2 pp  stock up  1 pp | **□**  **□**  **□**  **□**  **□**  **□**  **□** |  |
|  | - Respiratory masks approved for Mycobacteria exposure (N95 or higher efficiency) & replacement masks - Powered air-purifying respirators (PAPRs) & charged exchange batteries | 2 pp  as needed | **□**  **□** |  |
|  | - *Chainsaw operators*: Approved cutting-protective trousers and shoes, ear protection | as needed | **□** |  |
| **Dissection/Transport** | - Crane (hoist/forklift truck) with scale - Heavy chains, ropes, or straps - Hand or companion meat hooks with handles - Wheelbarrow(s)/roller carts - Large plastic tubs (concrete mixing ponds) - Engine-powered saw (reciprocal saw or chainsaw) with replacement chain/saw blade, fuel, and appropriate protective gear - Axes, cleavers, hammers, chisels, handsaws - Shovels or large dustpans - Water hose(s) | 1  10  6  6  6  1  à 2  2  1-2 | **□**  **□**  **□**  **□**  **□**  **□**  **□**  **□**  **□** |  |
| **Necropsy instruments** | - Large necropsy knives - Knife sharpener (steel) / belt grinder - Standard large animal necropsy instrument sets - Scalpel handles and replacement blades - Tape measure/folding rulers (2-5m) - Scales for organ weights,   *e.g.,* 0.01-3kg; 1-100kg   - Metal detector | 10  2/1  10  10  4  1  optional | **□**  **□**  **□**  **□**  **□**  **□**  **□** |  |
| **Documentation** | - Writing boards, pencils - Sampling forms/necropsy note forms - (Digital) camera with charged and replacement batteries, replacement memory card(s), appropriate illumination, size reference(s) (rulers), photo-pad (washable, smooth, non-reflecting surface, white or grey) | 6  as needed  1 | **□**  **□**  **□** |  |
| **Sampling** | - Disposable paper plates/bowels - Sterile instruments - Labels and waterproof marking pens - Plastic tubes for sample collection (screw cap) in 1ml, 2ml, 5ml, 12ml, 50ml, 200ml - Plastic buckets (5-10l) and - barrels (60l) with tight fitting lids - Culture swabs, sterile cups, glass slides - Serum tubes - Aluminum foil - Plastic molds/bowels and freezing mounting medium for cryohistology samples - Leak proof styrofoam boxes - Plastic bags (1-6l) - Formaldehyde solution, 4% neutral buffered - Glutaraldehyde solution, 3% neutral buffered - Modified Davidsons Fixative*** - Water ice - Dry ice - Liquid nitrogen - Isopentane | stock up  10  stock up stock up  10  2-3  stock up  stock up  stock up  stock up  stock up  stock up  10l  200ml  150ml  >20l  10l  8l  1l | **□**  **□**  **□**  **□**  **□**  **□**  **□**  **□**  **□**  **□**  **□**  **□**  **□**  **□**  **□**  **□**  **□**  **□**  **□** |  |
| **Waste disposal**  **& Disinfection** | - Biohazard bags (if legally required) - Approved tuberculocidal disinfectant - Scrubber / squeegee | stock up  stock up  2/2 | **□**  **□**  **□** |  |

*pers.=person

**pp=per person

*****Modified Davidson’s fixative solution** for eye histopathology: 30% of a 37-40% solution of formaldehyde, 15% ethanol, 5% glacial acetic acid, and 50% distilled H_2_O [44, 45]. Tissues are fixed for 24 h and then further processed or transferred into 4% neutrally buffered formaldehyde solution for another 12-24 h before further processing.
