# Supplementary material for: A pictural guide to postmortem examination of elephants
Source: PLoS One. 2026 Feb 9;21(2):e0338783. doi: 10.1371/journal.pone.0338783 (PMC12885571; doi:10.1371/journal.pone.0338783)
Supplement: S2 Table — (DOCX) [file pone.0338783.s006.docx]

**S2 Table. Personnel, necropsy teams and tasks.**

| **Team*** | | **Pers.**** | **Tasks*** |
| --- | --- | --- | --- |
| **Superintendent** | | 1 | - Person in charge for contact to the referring veterinarian(s), local authorities, and sample recipients. - Final responsibility for the safe, successful and complete performance of the necropsy and sampling process. - Organization, delegation, and control of prearrangements. - Specification of tissue/organ sample lists. - Instruction of personnel and division of the staff into separate necropsy groups. - Monitoring of the compliance with the set safety regulations, adequate waste disposal and disinfection. - Safeguarding of correct   - sample processing and shipment.   - documentation and reporting of findings. |
| **First-aiders** | | 2 | - Qualified and present during the necropsy. |
| **Secretary**  (“*clean hands*”) | | 1 | - Taking down notes & findings, filling in forms. - Keeping an overall view on the state and progress of the necropsy process. - Support of other personnel with short errands and assistance (restock consumables, duct-tape gloves of other members, *etc.*). |
| **Photographer** | | 1 | - Appropriate photo documentation of gross findings. |
| **Stand-in/spare man** | | 1-2 | - Support of other necropsy teams on demand. |
| **Knife sharpener** | | 2 | - Constant re-supply of sharp knives. |
| **Dismemberment**  **Team** | | 2-3 | - Experienced necropsy assistants or pathologists, crane and chainsaw operators. - Transport and dissection of the body, evisceration of body cavities. |
| **Organ system Teams** | **Locomotion** | 2-3 | - Dissection/removal and examination of the respective organ system/body parts. - Identification of alterations and arrangement of their photo documentation and sampling. - Reporting of findings to the head/secretary. - Taking of scheduled standard tissue samples. |
|  | **Gastrointestinal** | 2-3 |  |
|  | **Thorax** | 1-2 |  |
|  | **Head** | 1-2 |  |
|  | **Uro-genital** | 1-2 |  |
| **“Sampling”**  (“*clean hands*”) | | 2 | - Documentation of sampled tissues and taken specimens (list). - Appropriate labeling and packing of samples. - Taking of sterile samples from lesions. - Cutting and further processing of standard tissue samples for different downstream analyses:   - Histology   - Electron microscopy   - Cryohistology & molecular analyses (freezing in liquid nitrogen/dry ice). - Further processing of histology and electron microscopy samples (later). |
| **“Waste and disinfection”** | | 1-2 | - Safe and effective waste disposal and disinfection. |

*Combine multiple tasks appropriately as by the defined sequence of processes. *E.g.,* the head can simultaneously function as the secretary and the photographer; the members of the “Dismemberment team” will be allocated to one or more of the different organ system teams, subsequent to the completion of the dismemberment of the elephant body. **Except for the head, the indicated numbers of persons (Pers.) are minimal numbers.
