# Supplementary material for: A pictural guide to postmortem examination of elephants
Source: PLoS One. 2026 Feb 9;21(2):e0338783. doi: 10.1371/journal.pone.0338783 (PMC12885571; doi:10.1371/journal.pone.0338783)
Supplement: S3 Table — (DOCX) [file pone.0338783.s007.docx]

**S3 Table. Sampling and processing of tissue specimens**.

| **Sample types** | **Sample processing** |
| --- | --- |
| **Microbiology**  (Bacterio-/Viro-/Mycology) | Take sterile culture swabs before sampling or excise fresh tissue samples with sterile instruments and place in sterile culture dishes. For Tuberculosis testing, take multiple tissue samples for cell culture and PCR. |
| **Parasitology** | Take appropriate volume of fresh tissue/ingesta (>200 g) and fill in sterile sample container. |
| **Toxicology** | Take appropriate volume of fresh tissue/ingesta (500 ml/500 g) or vitreous (10 ml) and fill in sterile sample container. Take duplicate samples. Store at room temperature or refrigerate/freeze according to the scheduled downstream toxicology analyses. |
| **Histology** | Fix tissue samples (max. 2 cm thick) in neutrally buffered 4% formaldehyde solution (volume tissue: fixative = 1:10). If necessary, lamellate thick tissue/organ samples prior to fixation and change fixative solution after few hours. |
| **Electron microscopy** | Fix tissue pieces (max. 2 x 2 x 2 mm³) in 3% buffered glutaraldehyde solution for 6-12 hours (volume tissue: fixative = 1:10). |
| **Cryohistology** | Block tissue samples (10 x 5 x 5 mm³) in mounting medium in freezing bowls and freeze in liquid-nitrogen-cooled isopentane (-150°C), store at -80°C. |
| **Molecular/biochemical analysis** | Use sterile instruments to cut out multiple 2 x 2 x 2 mm³ tissue pieces from each sample. Freeze samples on dry ice, place in cryo-cup and store at -80°C. For long-term storage of samples, store at -150°C. |
