# Supplementary material for: A pictural guide to postmortem examination of elephants
Source: PLoS One. 2026 Feb 9;21(2):e0338783. doi: 10.1371/journal.pone.0338783 (PMC12885571; doi:10.1371/journal.pone.0338783)
Supplement: Attachment — Submitted filename: Response to Reviewers.pdf [file pone.0338783.s010.pdf]

To the editorial office of *PLOS ONE*

Munich, 20th of November, 2025

Dear Academic Editor Pierre Roques and Reviewers Francisco Pedraza-Ordoñez and Quentin Pascal,

We would like to sincerely thank you for taking the time to review and provide feedback on our manuscript (Research Article) entitled “A pictural guide to postmortem examination of elephants” by Falkenau *et al.* We greatly appreciate your careful consideration of this comprehensive work.

We have addressed the minor revision points to the best of our ability. Specifically:

- A grammar and spelling check was conducted throughout the manuscript, including the correction in line 430 (“und” → “and”, now in line 433), also throughout the S1-3 Material.
- References in the main manuscript as well as references in S3 Material have been formatted according to PLOS ONE requirements.
- No substantive changes to the content were made.

Please find attached the tracked and untracked revised versions of the manuscript for your review.

- All figures were checked with PACE and quality adjustments were made to Fig 11 and S1_Fig.

We hope that these revisions meet with your satisfaction, and we look forward to your feedback.

Thank you once again for your time and consideration.

Sincerely,

Dr. med. vet. Almuth Falkenau
Institute of Veterinary Pathology at the Center for Clinical Veterinary Medicine, LMU München
Veterinärstraße 13, 80539 Munich, GERMANY
Tel.: +49-(0)89-2180-2586
Email: Almuth.Falkenau@patho.vetmed.uni-muenchen.de
